# Evidence-based clinical practice guidelines for peptic ulcer disease 2026

**DOI:** 10.1007/s00535-026-02504-3

**Published:** 2026-08-24

**Authors:** Mitsushige Sugimoto, Tomoari Kamada, Masanori Ito, Junichi Iwamoto, Tadayoshi Okimoto, Shoko Ono, Takeshi Kanno, Toshimi Chiba, Kengo Tokunaga, Sachiyo Nomura, Takahisa Murao, Toshio Watanabe, Daisuke Asaoka, Eri Iwata, Ting Wang, Hideki Ono, Ryo Katsumata, Satoshi Kikuchi, Naoki Konishi, Satoshi Sato, Aya Sunago, Hiromi Sekiguchi, Hourin Cho, Daisuke Chinda, Osamu Dohi, Akinobu Nakata, Akira Higashimori, Yuka Hirashita, Keigo Misawa, Kazuhiro Mizukami, Daisuke Miyamori, Junko Mukohyama, Hideki Mori, Shuhei Yoshida, Jun Watanabe, Kiichi Satoh, Yuji Mizokami, Atsushi Takagi, Yoshifumi Takeyama, Susumu Eguchi, Sumio Watanabe, Hajime Isomoto, Takao Itoi, Satoshi Mochida

**Affiliations:** 1 Guidelines Committee for Creating and Evaluating the ‘‘Evidence-Based Clinical Practice Guidelines for Peptic Ulcer 2026 (4th Edition),” the Japanese Society of Gastroenterology (JSGE), Minato-ku, Tokyo, 105-0004 Japan; 2 Division of Genome-Wide Infectious Microbiology, Research Center for GLOBAL and LOCAL Infectious Disease, Oita University, 1-1 Idaigaoka, Hasama, Yufu, Oita 879-5593 Japan

**Keywords:** Peptic ulcer disease, *Helicobacter pylori*, Nonsteroidal anti-inflammatory drugs, Aspirin, Idiopathic ulcer, Vonoprazan

## Abstract

In 2026, the fourth edition of the evidence-based clinical practice guidelines for peptic ulcer disease by the Japanese Society of Gastroenterology (JSGE) was revised. These revised guidelines cover epidemiology, hemorrhagic peptic ulcers, *Helicobacter pylori* eradication therapy, non-eradication therapy, drug-induced ulcers, non*-H. pylori*, and nonsteroidal anti-inflammatory drug (NSAID) ulcers, remnant gastric ulcers, surgical treatment, and conservative therapy for perforation and stenosis. Although *H. pylori* infection and NSAIDs use remain two major causes of peptic ulcers, the prevalence and mortality rates of peptic ulcer have been declining due to the widespread use of eradication therapy and development of potent acid-inhibitory drugs in recent years. However, an increasing trend in idiopathic ulcers and ulcers caused by non-*H. pylori Helicobacter* (NHPH) infection has attracted attention. In this revised edition, therapeutic algorithms for the treatment of peptic ulcers based on *H. pylori* infection, low-dose aspirin (LDA) or NSAID use, ulcer history, and ulcer complications (e.g., hemorrhage) remain largely unchanged. This edition shows options for *H. pylori* eradication in various situations compared with the previous edition, and recommends the most effective regimens, regardless of Japanese insurance coverage. In addition, a new flowchart outlining the eradication treatment process has been presented. Compared with the previous version, the main changes in pharmacological treatment have been made: due to its acid-inhibitory characteristics compared with proton pump inhibitors and increased evidence of its efficacy and safety, vonoprazan has now been selected as the first-line treatment for peptic ulcers and NSAID- and LDA-induced ulcers.

## Introduction

In Japan, the number of patients with peptic ulcers has been declining annually. By 2023, the number of patients with gastric ulcers had fallen to 194,000 per year, and the number of patients with duodenal ulcers had fallen to 20,000 per year—a tenfold and twentyfold decrease, respectively, compared with the number of patients 40 years ago (https://www.mhlw.go.jp/toukei/saikin/hw/kanja/23/index.html). This downward trend can be attributed to the expanded indications under the national health insurance system for *H. pylori* eradication therapy, introduction of vonoprazan (VPZ), and wider primary and secondary prevention for drug-induced peptic ulcer [1].

Since the Japanese Society of Gastroenterology (JSGE) developed evidence-based clinical practice guidelines for peptic ulcer disease in 2009, the guidelines have been revised three times, with this version being the fourth edition [2]. These guidelines consist of clinical questions (CQs), background questions (BQs), and future research questions (FRQs) that are organized into nine categories. The guidelines, which were revised in the Japanese version as the fourth edition, include 25 CQs, 15 BQs, and 7 FRQs, whereas the English version includes 25 CQs and 4 FRQs (all CQs and selected 4 FRQs).

A literature search of the MEDLINE, the Cochrane Library, and Igaku Chuo Zasshi databases was performed to identify studies related to the CQs published between 1983 and January 2025. Evidence was evaluated using the Grading of Recommendations Assessment, Development and Evaluation (GRADE) system [3]. The quality of evidence was graded as A (high), B (moderate), C (low), and D (very low). Recommendation strength was classified as either ‘‘strong recommendation’’ or ‘‘weak recommendation.’’ The systematic review (SR) team conducted meta-analysis by mainly using randomized controlled trials (RCTs) and determined the overall strength of the evidence from A to D. A recommendation was classified as “strong recommendation” when ≥70% of votes supported a strong recommendation and as “weak recommendation” when ≥70% supported either a strong or weak recommendation.

## Hemorrhagic gastric and duodenal ulcers

### Endoscopic hemostatic therapy

#### CQ-1

What acid-suppressive agents are useful after endoscopic hemostasis for hemorrhagic peptic ulcers?

- Proton-pump inhibitors (PPIs) are recommended after endoscopic hemostasis for hemorrhagic peptic ulcers.

Recommendation: strong (100% agreed for strong recommendation), evidence level: A.

- VPZ is suggested after endoscopic hemostasis for hemorrhagic peptic ulcers.

Recommendation: weak (16.7% agreed for strong, 83.3% agreed for weak recommendation), evidence level: A.

*Comments:* As stated in the 2020 guidelines [2], PPI administration after endoscopic hemostasis reduces rebleeding rates, transfusion requirements, the duration of hospitalization, and need for surgery.

Previous meta-analyses showed similar efficacy across PPI doses and administration routes [4–6].

Two RCTs comparing VPZ 40 mg with high-dose continuous PPI (pantoprazole) infusion—an 80 mg bolus followed by continuous infusion at 8 mg/hour for 72 h—have been reported [7, 8]. No differences were observed in rebleeding rates or outcomes such as mortality, need for additional treatment, or hospital stay. VPZ may prevent rebleeding similarly to PPI therapy, but these findings require caution because both drugs were used at high doses.

### *Helicobacter pylori* eradication therapy

When *H. pylori* infection is treated with eradication therapy, the regimen should maximize eradication and minimize adverse events. The prevalence of drug-resistant strains varies by region and treatment period [9].

A meta-analysis has reported that pretreatment with acid-inhibitory drugs does not affect the success of eradication therapy [10]. Therefore, although acid-inhibitory drugs are recommended after endoscopic hemostasis for hemorrhagic peptic ulcers, it is appropriate to perform *H. pylori* eradication therapy early following endoscopic hemostasis, irrespective of during PPI administration.

### First-line eradication therapy

#### CQ-2

Does VPZ improve eradication rates compared with PPIs?

- Since a triple regimen containing VPZ achieves a higher eradication rate than regimens using PPIs in the first-line eradication therapy, the use of VPZ is recommended.

Recommendation: strong (100% agreed for strong recommendation), evidence level: A.

- Since the eradication rates and incidence of adverse events are comparable between the VPZ–amoxicillin (AMPC)–metronidazole (MNZ) regimen and the PPI–AMPC–MNZ regimen during second-line eradication therapy, either a PPI or VPZ is recommended when using AMPC-MNZ.

Recommendation: weak (66.7% agreed for strong, 33.3% agreed for weak recommendation), evidence level: B.

**Fig. 1:**
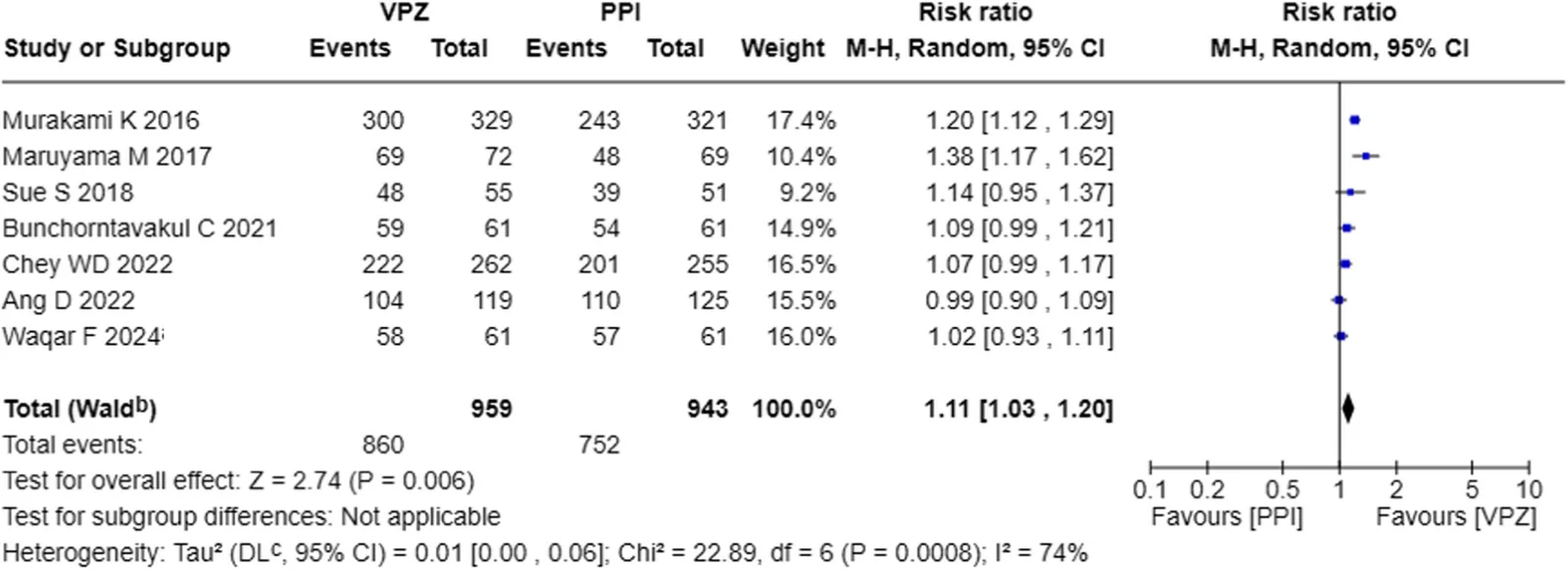
Comparison of eradication rates in the ITT analysis between VPZ-containing and PPI-containing triple regimens in first-line eradication therapy using RCTs. *CI* confidence interval, *DL* DerSimonian–Laird, *ITT* intention-to-treat, *M-H* Mantel–Haenszel, *PPI* proton pump inhibitor, *RCTs* randomized controlled trials, *VPZ* vonoprazan

*Comment:* Potent acid inhibition is important for successful eradication [11]. VPZ inhibits acid secretion more rapidly and potently than PPIs [12]. A meta-analysis of seven RCTs [13, 14] found that VPZ therapy achieved a higher eradication rate than PPI therapy, particularly in patients infected with clarithromycin (CAM)-resistant strains (Fig. 1). For second-line eradication therapy [15], eradication rates were comparable between the VPZ–AMPC–MNZ and PPI–AMPC–MNZ regimens.

Recommended regimens:

- First-line eradication therapy: VPZ-containing eradication regimen.
- Second-line eradication therapy: PPI (standard dose) or VPZ (20 mg) + AMPC (750 mg) + MNZ (250 mg) twice daily (bid) for 1 week.

#### CQ-3

Is CAM susceptibility testing recommended before first-line eradication therapy?

- If possible, CAM susceptibility testing should be performed before first-line eradication therapy, and the regimen expected to achieve the highest eradication rate should be selected.

Recommendation: strong (100% agreed for strong recommendation), evidence level: A.

*Comments:* CAM resistance is a critical factor influencing the outcomes of *H. pylori* eradication therapy. In Japan, the CAM-resistance rate remains high at 35.5% [16]. A meta-analysis by Ma et al. [17] confirmed that susceptibility-guided individualized regimens achieve significantly higher eradication rates than empirical regimens. Nucleic acid amplification tests can detect 23S rRNA mutations from gastric fluid rapidly and accurately, enabling rapid tailored therapy [18].

#### CQ-4

Which regimen is recommended when CAM susceptibility testing has been performed?

- For CAM-susceptible strains, VPZ–AMPC–CAM therapy is recommended.
- Recommendation: strong (100% agreed for strong recommendation), evidence level: A.
- For CAM-resistant strains, PPI- or VPZ-based therapy with AMPC and MNZ is recommended.
- Recommendation: strong (91.7% agreed for strong recommendation); evidence level: A.

*Comments:* In Japan, the standard first-line *H. pylori* eradication regimen consisting of VPZ–AMPC–CAM achieves an eradication rate exceeding 90%; however, its efficacy is significantly reduced in patients infected with CAM-resistant strains [13]. A meta-analysis of RCTs demonstrated that CAM susceptibility-guided therapy significantly improved eradication rates [19]. Therefore, VPZ–AMPC–CAM regimen is recommended when CAM susceptibility is confirmed.

In the same meta-analysis [19], PPI–AMPC–MNZ, high-dose PPI–AMPC, and PPI–AMPC–levofloxacin (LVFX) were evaluated as susceptibility-guided treatment regimens for patients with CAM-resistant strains in randomized controlled trials. PPI–AMPC–MNZ is recommended based on consistent evidence of efficacy from multiple RCTs [20–23].

Recommended regimens:

- CAM-susceptible strains: VPZ (20 mg) + AMPC (750 mg) + CAM (200 mg) bid for 1 week.
- CAM-resistant strains: PPI (standard dose) or VPZ (20 mg) + AMPC (750 mg) + MNZ (250 mg) bid for 1 week.

#### CQ-5

Which regimen is recommended if CAM susceptibility testing is unavailable for first-line therapy?

- When CAM susceptibility testing is unavailable for first-line therapy, either VPZ–AMPC–CAM or PPI–AMPC–MNZ is recommended.

Recommendation: strong (100% agreed for strong recommendation), evidence level: A.

*Comments:* In the meta-analysis by Liu et al. [24], the VPZ–AMPC–CAM regimen demonstrated a higher eradication rate than the PPI–AMPC–CAM regimen. In areas with low MNZ and high CAM resistance, such as Japan, the PPI–AMPC–MNZ regimen was superior to the PPI–AMPC–CAM regimen [25]. However, evidence regarding the efficacy of the VPZ–AMPC–MNZ regimen remains insufficient. Therefore, VPZ–AMPC–CAM is recommended and is covered by Japanese health insurance.

Recommended regimens:

- VPZ (20 mg) + AMPC (750 mg) + CAM (200 mg) bid for 1 week.
- PPI (standard dose) + AMPC (750 mg) + MNZ (250 mg) bid for 1 week.

#### CQ-6

Should dual therapy with VPZ and AMPC be recommended?

- Since the VPZ-AMPC regimen has a lower eradication rate than the VPZ–AMPC–CAM regimen as first-line therapy, we recommend against selecting the dual regimen as the first-line therapy.

Recommendation: weak (33.3% agreed for strong, 66.7% agreed for weak recommendation), evidence level: A.

**Fig. 2:**
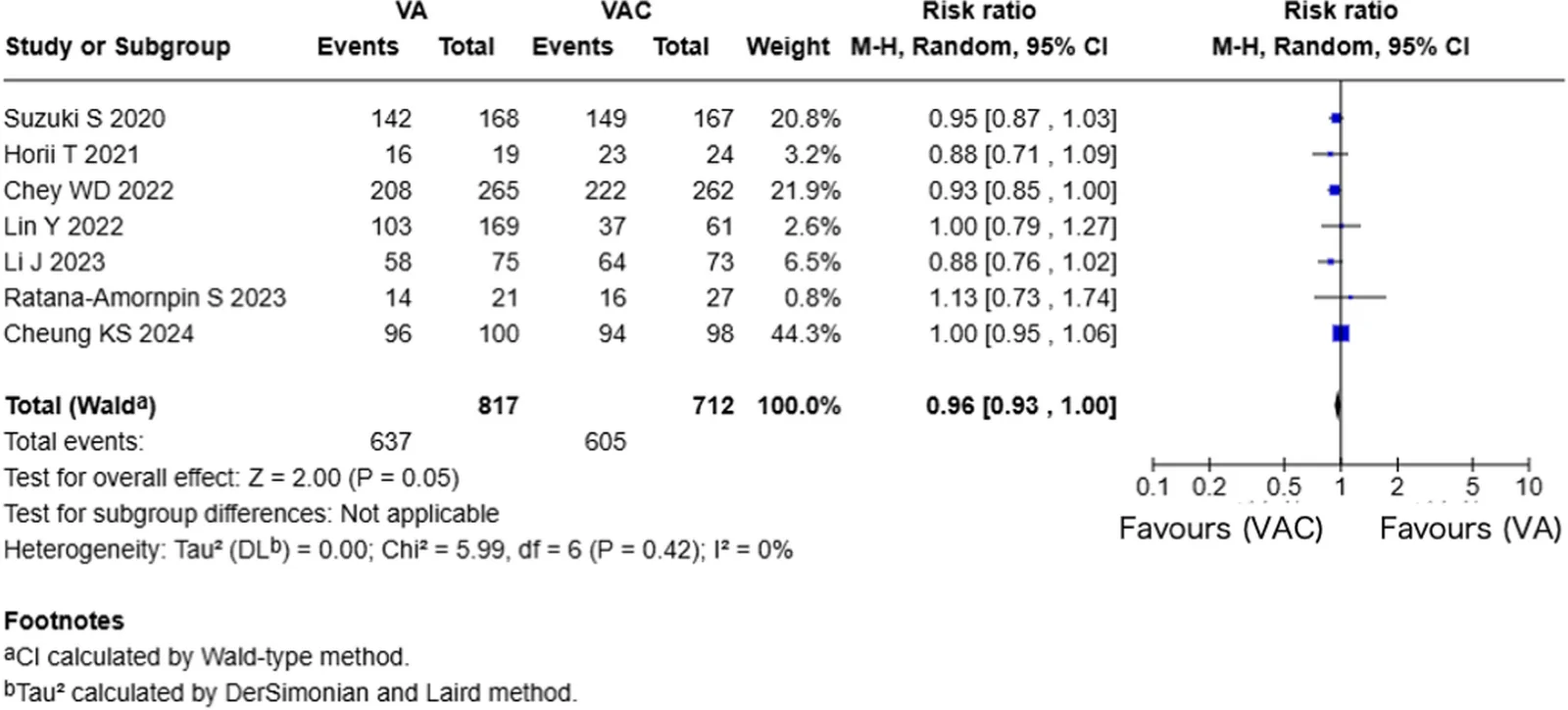
Comparison of eradication rates in the ITT analysis between VPZ–AMPC therapy and VPZ–AMPC–CAM therapy in first-line eradication therapy using RCTs. *AMPC* amoxicillin, *CAM* clarithromycin, *CI* confidence interval, *DL* DerSimonian–Laird, *ITT* intention-to-treat, *M-H*, Mantel–Haenszel, *RCTs* randomized controlled trials, *VA* VPZ plus AMPC, *VAC* VPZ plus AMPC and CAM, *VPZ* vonoprazan

*Comment:* The prevalence of CAM-resistant strains in Japan has reached 35–40% [16]. Recently, international guidelines recommend VPZ–AMPC dual therapy as a candidate regimen [9]. A meta-analysis of seven RCTs [26, 27] found that VPZ–AMPC dual regimen had a significantly lower rate than the triple regimen (Fig. 2). There are no reports of RCTs evaluating the efficacy and safety of VPZ–AMPC therapy as second-line or third-line treatment. When dual therapy is selected, AMPC dosing frequency should be increased to maintain therapeutic plasma concentrations [28].

Recommended regimens:

- First-line eradication therapy: VPZ (20 mg) + AMPC (750 mg) + CAM (200 mg) bid for 1 week.

### Second-line eradication therapy

#### CQ-7

Which regimen is recommended for second-line *H. pylori* eradication therapy after use of CAM in first-line therapy?

- Triple therapy with a PPI or VPZ–AMPC–MNZ for 7 days is recommended.

Recommendation: strong (91.7% agreed for strong, 8.3% agreed for weak recommendation), evidence level: B.

*Comment:* According to the only small-scale RCT, 7-day VPZ–AMPC–MNZ showed eradication rates comparable to rabeprazole-based therapy [15]. A meta-analysis of 16 observational studies reported that the VPZ group had a higher eradication rate than the PPI group (odds ratio [OR]: 1.51, 95% CI: 1.27–1.81, p < 0.001) [29]. Regarding safety, no clear difference was observed between VPZ and PPI in RCTs [15]. Overall, VPZ-based second-line therapy showed similar efficacy and safety to standard PPI therapy, but evidence for clear superiority remains limited.

Recommended regimens:

- PPI (standard dose) or VPZ (20 mg) + AMPC (750 mg) + MNZ (250 mg) bid for 1 week.

### Third-line eradication therapy

#### CQ-8

Which regimen is recommended after the failure of MNZ-based second-line eradication therapy?

- Triple therapy with a PPI or VPZ–AMPC–sitafloxacin (STFX) is suggested (not covered by the Japanese insurance system).

Recommendation: weak (33.3% agreed for strong, 66.7% agreed for weak recommendation), evidence level: B.

*Comments:* We conducted a network meta-analysis using RCTs evaluating five regimens [30–34] that met our inclusion criteria: (1) PPI–AMPC–STFX, (2) VPZ–AMPC–STFX, (3) PPI–MNZ–STFX, (4) PPI–AMPC, and (5) PPI–AMPC–LVFX. Compared with PPI–AMPC–STFX, VPZ–AMPC–STFX showed the largest effect size, although the difference was not statistically significant.

Although there has been an increase in antimicrobial agent-resistant *H. pylori* strain in clinical practice, the introduction of VPZ and selection of appropriate drugs has led to a decrease in the number of patients where eradication is not achieved by the second-line eradication therapy. Among patients with failed second-line eradication, when assessed using the urea breath test (UBT), false-positive results may occur due to the influence of urease-producing non-*H. pylori* bacteria, particularly in conditions such as autoimmune gastritis with severe atrophy. Therefore, careful consideration for needs of additional therapy is required before initiating third-line or subsequent rescue salvage therapies.

Recommended regimens:

- VPZ (20 mg) or PPI (standard dose) + AMPC (750 mg) + STFXZ (100 mg) bid for 1 week.

#### FRQ-1

What is “endless eradication”? Are there any precautions in these cases?

“Endless eradication” refers to the medical practice of adding unnecessary eradication treatments despite successful eradication therapy due to false-positive results on the UBT. This is often seen in cases of autoimmune gastritis and requires careful attention.

*Comment:* The term “endless eradication” is a concept reported by Furuta et al. [35]. After successful eradication, reduced gastric acid secretion may allow urease-producing non-*H. pylori* bacteria to colonize the stomach, causing weakly positive ^13^C-UBT results and unnecessary repeated treatment. In cases of autoimmune gastritis, gastric acid secretion remains reduced even after successful eradication, increasing the risk of getting endless eradication. Furuta et al. reported that 18% of cases in which eradication therapy was unsuccessful two or more times were autoimmune gastritis [35].

### Others

#### CQ-9

Which regimen is recommended for patients with penicillin allergy?

- A regimen combining VPZ or PPI–MNZ–STFX, or VPZ–MNZ–CAM, is suggested.

Recommendation: weak (16.7% agreed for strong, 83.3% agreed for weak recommendation), evidence level: D.

*Comments:* For patients with penicillin allergy, RCTs using tetracycline or bismuth have been reported overseas. Evidence in Japan is limited to small observational studies; however, 7-day VPZ-based regimens (VPZ–MNZ–STFX and VPZ–MNZ–CAM) showed high eradication rates [36–38]. A 7-day PPI–MNZ–STFX regimen was also effective, whereas PPI–MNZ–CAM showed poor efficacy [39, 40].

Recommended regimens:

- VPZ (20 mg) or PPI (standard dose) + MNZ (250 mg) + STFX (100 mg) bid for 1 week.
- VPZ (20 mg) + MNZ (250 mg) + CAM (200 mg) bid for 1 week.

#### CQ-10

Which regimen is recommended for patients with liver dysfunction?

- For first-line eradication, a regimen combining PPI–AMPC–CAM or MNZ is suggested. For second-line eradication, a regimen combining PPI–AMPC–MNZ is suggested.

Recommendation: weak (25.0% agreed for strong, 75.0% agreed for weak recommendation), evidence level: C.

*Comments:* No clinical trials have directly compared eradication regimens in patients with liver dysfunction. However, a meta-analysis of three observational studies comparing patients with hepatic impairment to healthy individuals demonstrated that eradication rates were not significantly lower in the former group [41–43]. Adverse-event rates also did not increase significantly. Although evidence is limited, standard first- and second-line regimens may be used with careful monitoring of hepatic function and drug metabolism.

Recommended regimens:

- First-line eradication therapy: PPI (standard dose) + AMPC (750 mg) + CAM (200 mg) or MNZ (250 mg) bid for 1 week.
- Second-line eradication therapy: PPI (standard dose) + AMPC (750 mg) + MNZ (250 mg) bid for 1 week.

#### CQ-11

Which regimen is recommended for patients with renal dysfunction or those on hemodialysis?

- For non-dialysis patients with renal impairment, an AMPC-free regimen, such as a combination of a PPI–CAM–MNZ, is suggested when the benefits of eradication are considered significant.

Recommendation: weak (16.7% agreed for strong, 83.3% agreed for weak recommendation), evidence level: C.

- For patients on hemodialysis, we suggest dose-reduced regimens such as a PPI–AMPC–MNZ or PPI–AMPC–CAM. Precise dosage adjustments should be determined in consultation with renal specialists.

Recommendation: weak (33.0% agreed for strong, 66.7% agreed for weak recommendation), evidence level: C.

**Fig. 3:**
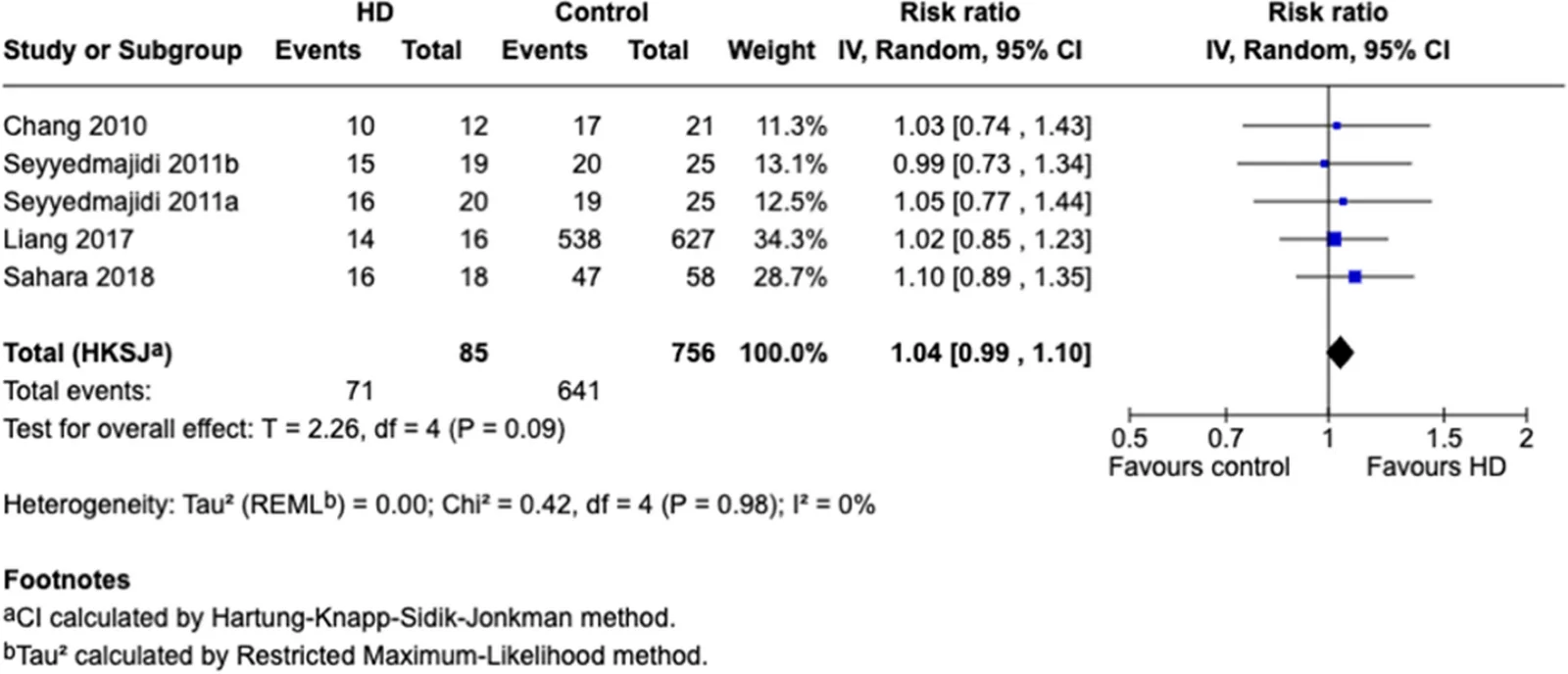
Eradication rates in healthy participants receiving standard doses and hemodialysis patients receiving reduced-dose regimens in observational studies. *CI* confidence interval, *HD* hemodialysis, *HKSJ* Hartung–Knapp–Sidik–Jonkman, *IV* inverse variance, *REML* restricted maximum likelihood

*Comments:* In non-dialysis patients with renal impairment, one randomized controlled trial demonstrated that a regimen of lansoprazole–CAM–MNZ was more effective and safer than AMPC-based triple therapy [44]. For patients on hemodialysis, a meta-analysis of observational studies confirmed that dose-reduced regimens achieve eradication rates equivalent to those observed in healthy individuals (Fig. 3) [45–48], while minimizing minor adverse events, including dysgeusia or abdominal distension.

Recommended regimens:

- Non-hemodialysis patients: PPI + CAM + MNZ or non-AMPC regimen.
- Hemodialysis patients: PPI + AMPC (reduced) + CAM or MNZ.
  - AMPC dose: 250–375 mg per dose, bid.
  - Hemodialysis in the morning: post-hemodialysis and evening time, bid.
  - Hemodialysis in the evening: morning time and post-hemodialysis, bid or post-hemodialysis (500 mg) once daily (oid).

#### CQ-12

What is the upper age limit for eradication therapy in older patients with peptic ulcer disease?

- Eradication therapy is suggested for older patients with peptic ulcer disease regardless of age because it may help prevent ulcer recurrence in the short term.

Recommendation: weak (33.3% agreed for strong, 66.7% agreed for weak recommendation), evidence level: D.

*Comments:* In an observational study, among patients aged ≥65 years who underwent eradication therapy for peptic ulcer disease, successful eradication reduced 1-year ulcer recurrence [49]. Several studies reported no difference in eradication rates between older and younger patients [50]. Although age ≥80 years was reported as an independent factor for eradication failure, this difference disappeared after second-line therapy [51]. Adverse events did not significantly increase, suggesting good tolerability in considerably older patients [52]. Therefore, eradication therapy in older patients is suggested for preventing ulcer recurrence, with careful consideration of comorbidities.

#### FRQ-2

How does* H. pylori* eradication therapy affect gut microbiota?

- Eradication therapy temporarily reduces the diversity of the gut microbiota, but it gradually recovers over time. However, the time it takes for diversity to recover varies depending on factors such as the dosages and duration of the combined acid suppressants and antibiotics.

*Comments:* Eradication therapy temporarily reduces gut microbiota diversity, but recovery typically occurs over time. A meta-analysis by Ye et al. [53] indicated a short-term decrease in α-diversity, though long-term data remained inconclusive due to variables such as antibiotic types, dosages, and treatment duration. In Japan, Horii et al. [54] found that VPZ–AMPC–CAM significantly reduced α-diversity at 1 and 8 weeks, whereas VPZ–AMPC showed no significant change.

General findings from multiple RCTs [55, 56] suggest that diversity decreases within 2 weeks, trends toward recovery between 8 and 12 weeks, and is largely restored by 1 year. Furthermore, Bai et al. [57] highlighted that probiotic supplementation helps prevent dysbiosis and mitigates gastrointestinal side effects. Further research is needed to clarify the long-term effects of eradication therapy on the gut microbiota.

## Drug-induced ulcers

### Nonsteroidal anti-inflammatory drug (NSAID)-induced ulcer

#### Prevention

##### CQ-13

Is preventive therapy for NSAID-induced ulcers necessary in patients with no history of ulcers?

- PPIs are recommended for the prevention of NSAID-induced ulcers, even in patients without a history of peptic ulcers.

Recommendation: strong (100% agreed for strong, recommendation), evidence: level A.

**(**PPIs are not covered by Japanese health insurance for primary prevention of ulcers in patients taking NSAIDs)

*Comment:* In patients receiving NSAID therapy for more than 3 months, prostaglandin (PG) analogs [58], PPIs [59], and high-dose H_2_RAs [60] have shown preventive efficacy. Scally et al. [61] showed that PPIs had greater protective effects than H_2_RAs or PG analogs against peptic ulcers and further bleeding; therefore, PPIs are suggested as a first-line therapy.

##### CQ-14

What measures should be taken to prevent ulcer recurrence in patients with a history of peptic ulcers or bleeding ulcers who require NSAID therapy?

- VPZ or PPIs are recommended and VPZ is suggested for the prevention of ulcers in patients with a history of ulcers.

Recommendation: strong (100% agreed for strong recommendation), evidence: level B.

- Concomitant administration of a selective cyclooxygenase (COX)− 2 inhibitor with a VPZ or PPI is recommended for preventing recurrence of bleeding NSAID-induced ulcers in patients with a history of bleeding ulcers.

Recommendation: weak (33.3% agreed for strong, 66.7% agreed for weak recommendation), evidence level: B.

*Comment:* Although PG analogs [62] are effective in high-risk patients with a history of peptic ulcers, patient dropout owing to diarrhea is common.

**Fig. 4:**
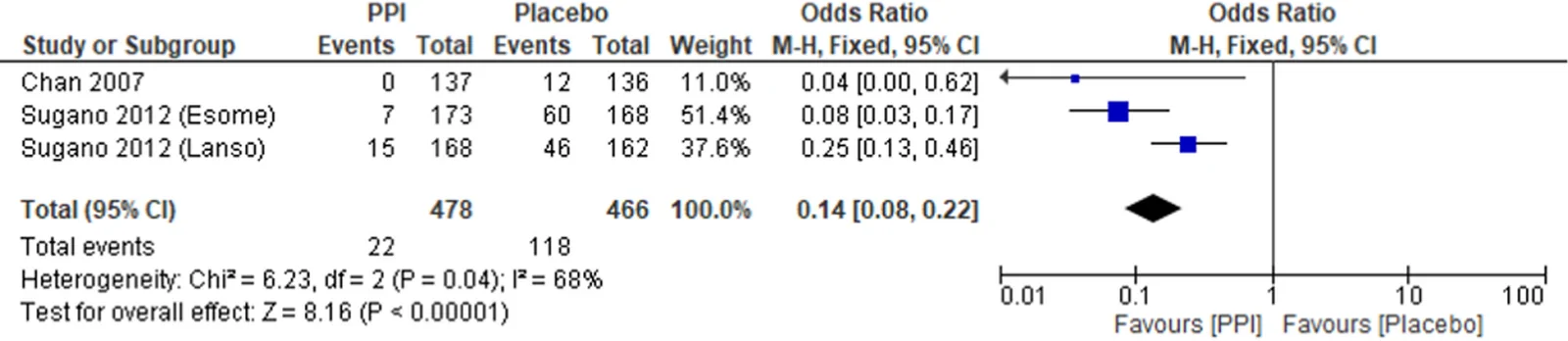
Meta-analysis of the preventive effect of secondary prevention of NSAIDs-induced ulcers. *CI* confidence interval, *Esome* esomeprazole, *Lanso* lansoprazole, *M-H* Mantel–Haenszel, *NSAID* nonsteroidal anti-inflammatory drug, *PPI* proton pump inhibitor

Studies have shown that PPIs were superior to placebo in reducing ulcer recurrence during long-term NSAID therapy [63, 64]. Therefore, PPIs are recommended as first-line therapy (Fig. 4).

Chan et al. suggested that combination therapy with a selective COX-2 inhibitor and PPI was more effective than a selective COX-2 inhibitor alone for preventing ulcer bleeding in high-risk patients [65].

Mizokami et al. [66] reported that VPZ was not inferior to PPIs, indicating that VPZ can be recommended for the secondary prevention of NSAID-induced peptic ulcer similar to PPIs. Prospective real-world data also support VPZ efficacy and safety during long-term NSAIDs therapy [67].

##### CQ-15

How should NSAID-induced ulcers be prevented in patients receiving concomitant NSAIDs and antithrombotic agents and/or glucocorticoids, and in those with severe comorbidities?

- In patients receiving NSAIDs in combination with glucocorticoids and/or antithrombotic drugs, the use of a COX-2 inhibitor is recommended for ulcer prevention.

Recommendation: strong (91.7% agreed for strong recommendation), evidence: level B.

- In patients with severe comorbidities, the use of VPZ or PPIs is recommended for the prevention of NSAID-induced ulcers.

Recommendation: strong (83.3% agreed for strong recommendation), evidence: level B.

*Comment:* Concomitant use of nonselective NSAIDs or low-dose aspirin (LDA), but not selective COX-2 inhibitors, with corticosteroids or anticoagulants increases the risk of upper gastrointestinal bleeding (UGIB), suggesting that selective COX-2 inhibitors may be associated with a lower risk of UGIB [68]. In high-risk patients with comorbidities, PPIs reduced a risk of serious NSAID-related ulcer complications [69]. VPZ is also effective in preventing ulcer recurrence during long-term NSAID therapy in patients with comorbidities [67].

## LDA-induced ulcer

### Treatment

#### CQ-16

How should LDA-related peptic ulcer be treated?

- Concomitant use of a PPI with continuous LDA therapy is recommended for LDA-related peptic ulcer.

Recommendation: strong (100% agreed for strong recommendation); evidence: level A.

*Comment*: For patients with a history of LDA-related peptic ulcer bleeding, continuous LDA therapy with a PPI after endoscopic hemostasis reduced cardiovascular mortality without increasing recurrent bleeding [70]. The peptic ulcer healing rate was comparable between a PPI alone and LDA combined with a PPI [71].

### Prevention

#### CQ-17

In patients without a history of peptic ulcer or peptic ulcer bleeding, is the prevention of LDA-related peptic ulcer necessary?

- It is necessary to prevent the development of ulcers because peptic ulcer bleeding in patients taking LDA affects their prognosis.
- A PPI is recommended for the primary prevention of LDA-related peptic ulcer in patients without a history of peptic ulcer or peptic ulcer bleeding.

Recommendation: strong (100% agreed for strong recommendation), evidence level: A.

- In patients receiving dual-antiplatelet drug therapy (DAPT), a PPI or VPZ is recommended for the primary prevention of UGIB in those without a history of peptic ulcer or peptic ulcer bleeding. (Not covered by Japanese insurance except for cases with a history of ulcers)

Recommendation: strong (91.7% agreed for strong recommendation), evidence level: A.

**(**PPI and VPZ are not covered by Japanese insurance for primary prevention of ulcers in patients taking LDA)

**Fig. 5:**
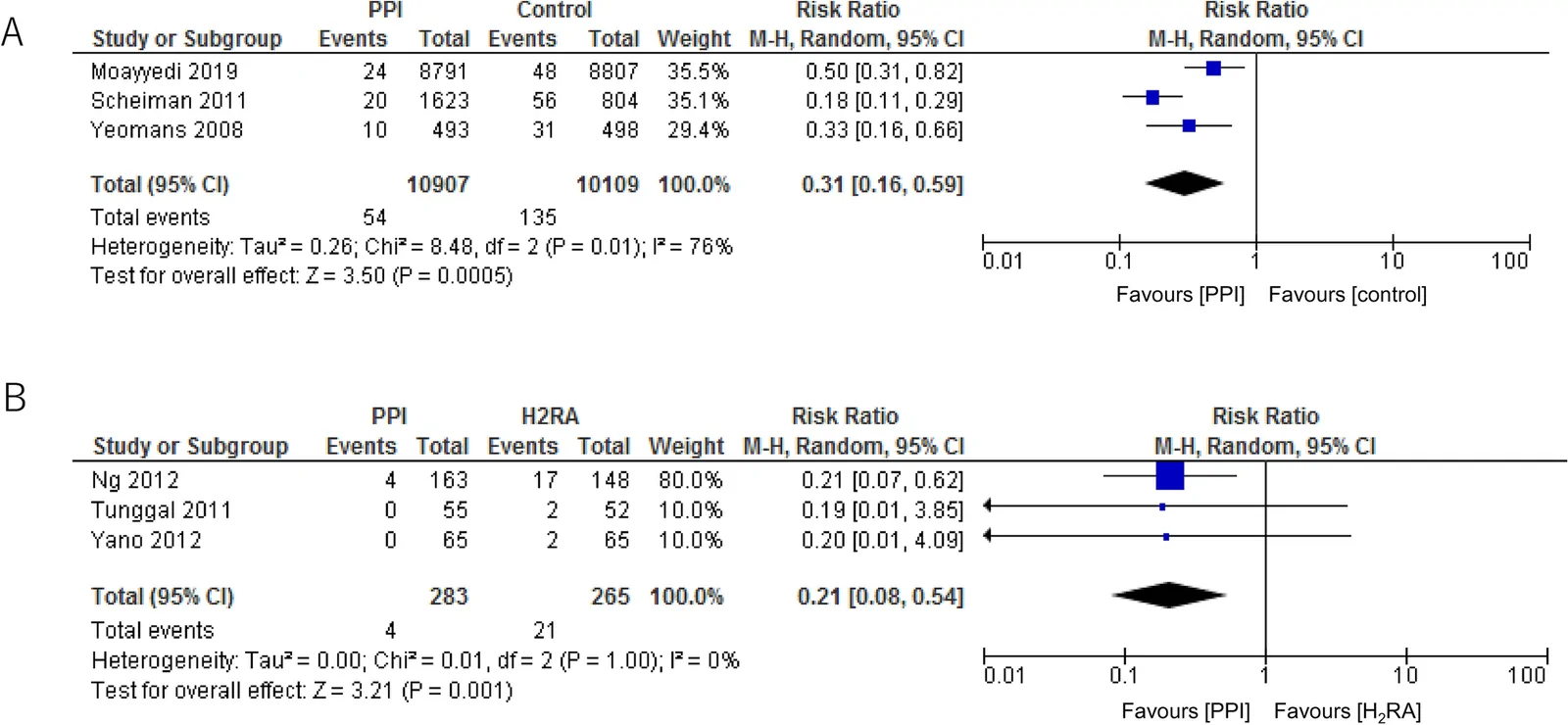
Efficacy of a PPI in LDA-related peptic ulcer without a history of peptic ulcer or peptic ulcer bleeding (**A**). A PPI showed a reduction in the risk of peptic ulcer development compared with placebo. Analysis of UGIB in patients receiving DAPT (**B**). A PPI significantly prevented the incidence of UGIB compared with an H_2_RA. *CI* confidence interval, *DAPT* dual-antiplatelet therapy, *H2RA* histamine H2-receptor antagonist, *LDA* low-dose aspirin, *M-H* Mantel–Haenszel, *PPI* proton pump inhibitor, *UGIB* upper gastrointestinal bleeding

*Comment:* A meta-analysis of the efficacy of PPIs in LDA-treated patients showed that a PPI reduced the risk of peptic ulcer occurrence compared with placebo [72–74] (Fig. 5A).

In DAPT, a meta-analysis of three studies [75–77] with 95%–100% of patients without a history of peptic ulcer showed that PPI significantly prevented UGIB compared with H_2_RA (RR: 0.21, 95% CI 0.08–0.54, *p* = 0.001) (Fig. 5B).

UGIB incidence did not differ significantly between VPZ and PPI in patients receiving LDA and thienopyridine antiplatelet agents [78].

#### CQ-18

How to prevent recurrence of LDA-related peptic ulcer in patients with a history of peptic ulcers?

- A PPI or VPZ is recommended for reducing the recurrence rate of LDA-related peptic ulcer.

Recommendation: strong (100% agreed for strong recommendation), evidence: level A.

**Fig. 6:**
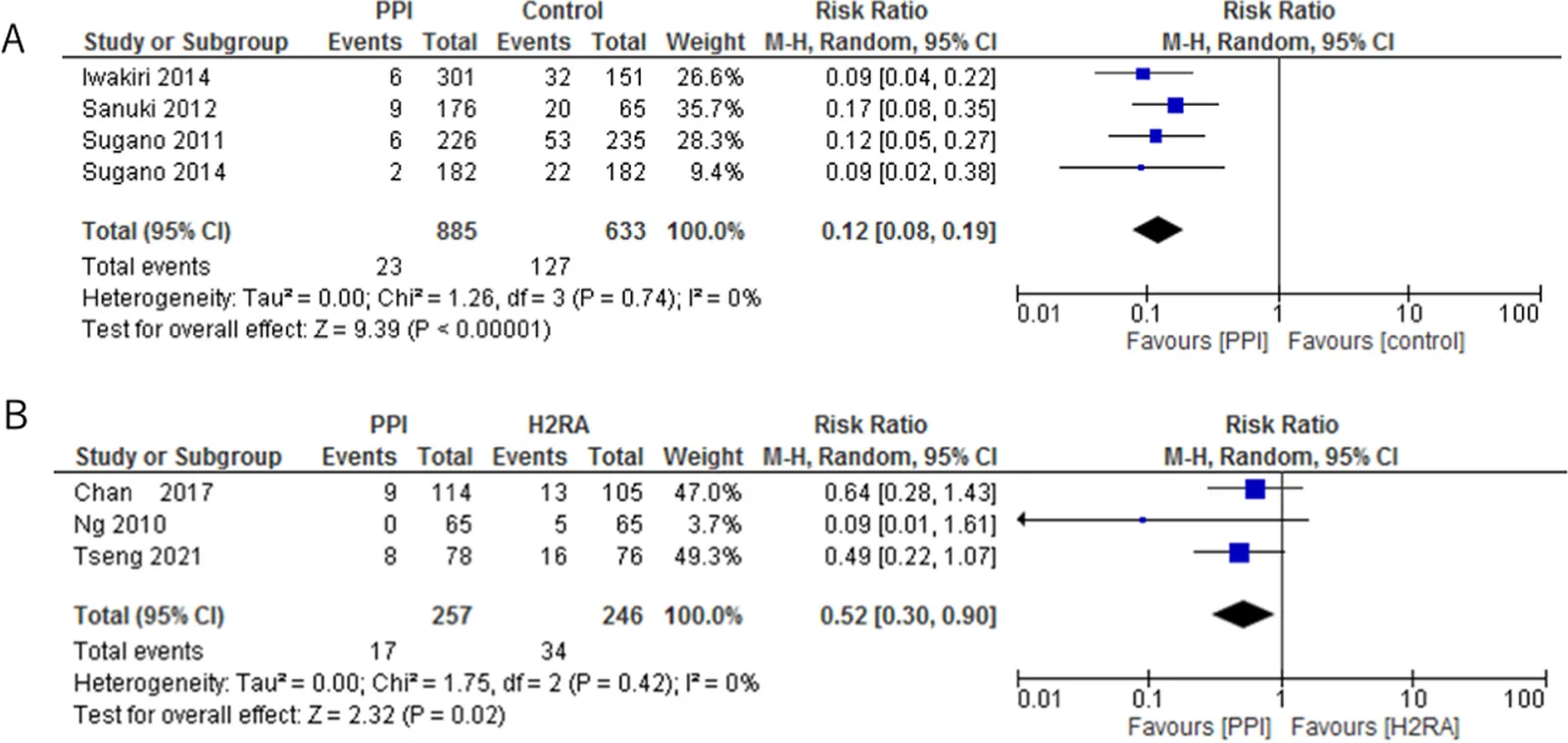
Peptic ulcer recurrence rate in patients continuing LDA with a history of peptic ulcers (**A**). A PPI reduced the rate of peptic ulcer recurrence compared with placebo or gefarnate. Peptic ulcer recurrence rate in patients continuing LDA with a history of peptic ulcers (**B**). A PPI significantly reduced peptic ulcer recurrence compared with an H_2_RA. *CI* confidence interval, *H*_*2*_*RA* histamine H2-receptor antagonist, *LDA* low-dose aspirin, *M-H* Mantel–Haenszel, *PPI* proton pump inhibitor

*Comment:* A meta-analysis of four RCTs investigating the efficacy of PPIs in reducing the rate of LDA-related peptic ulcer recurrence showed that a PPI reduced the rate of peptic ulcer recurrence compared with placebo or gefarnate [79–82] (Fig. 6A).

In a meta-analysis of three studies comparing PPIs and H_2_RAs [83–85], a PPI reduced ulcer recurrence compared with an H_2_RA (Fig. 6B).

When LDA-induced peptic ulcer recurrence was compared between VPZ and lansoprazole, VPZ 10 mg significantly inhibited recurrence compared with lansoprazole [86].

#### CQ-19

How to prevent rebleeding in patients taking LDA with a history of UGIB?

- A PPI is recommended to prevent recurrent UGIB in patients receiving LDA. In *H. pylori*-positive patients, a PPI is recommended after *H. pylori* eradication.

Recommendation: strong (100% agreed for strong recommendation), evidence: level B.

(PPI is not covered by Japanese insurance for secondary prevention of peptic ulcer bleeding in patients taking LDA)

*Comment*: The frequency of recurrence of LDA-related peptic ulcer bleeding was decreased by lansoprazole after *H. pylori* eradication [87]. In patients after *H. pylori* eradication or in *H. pylori*-negative patients with a history of peptic ulcer bleeding, a PPI in addition to continuous LDA treatment prevented UGIB compared with an H_2_RA [83]. Therefore, a PPI is recommended to prevent UGIB in patients taking LDA who are at risk of cardiovascular events.

#### CQ-20

What medications are useful in preventing ulcer recurrence when NSAIDs are used in patients taking LDA?

- Celecoxib with concomitant use of a PPI is recommended for the prevention of recurrence of peptic ulcers during NSAID treatment in patients taking LDA.

Recommendation: strong (91.7% agreed for strong recommendation), evidence: level A.

*Comment:* In patients with a history of peptic ulcer who are receiving combinations of NSAIDs and LDA, concomitant use of a PPI reduced the risk of gastric ulcer recurrence [88, 89]. Furthermore, patients receiving a COX-2 inhibitor plus LDA had fewer gastrointestinal events than those receiving a nonselective NSAID plus LDA [90, 91].

### Anticoagulants

#### CQ-21

What strategies are recommended to prevent UGIB in patients receiving anticoagulant therapy?

- In patients taking anticoagulants who are at high risk of bleeding, concomitant use of a PPI is suggested to prevent peptic ulcer bleeding.

Recommendation: weak (25.0% agreed for strong, 75.0% agreed for weak recommendation), evidence level: C.

*Comments:* In patients receiving anticoagulant therapy, concomitant use of PPIs significantly reduces the risk of UGIB. In particular, among patients with a history of UGIB, PPI co-therapy significantly reduced the risk of rebleeding [92].

Regarding individual anticoagulants, reduced UGIB risk with PPI co-therapy has been reported for warfarin and edoxaban [74, 93].

European guidelines also recommend PPI co-therapy for high-risk patients with a history of UGIB who require continued anticoagulant therapy [94].

### PPI/P-CAB-related adverse events

#### FRQ-3

Does long-term use of PPIs or VPZ lead to the development of gastric neoplasms or other mucosal lesions?

- Long-term use of PPIs or VPZ may be associated with the development of various gastric mucosal lesions; therefore, prolonged use should be undertaken with caution.

*Comments:* Long-term use of PPIs and VPZ may be associated with various gastric mucosal lesions, including benign and malignant tumors, and other mucosal changes. Proposed mechanisms include hypergastrinemia, gastric microbiota alterations, and direct drug effects. Meta-analyses [95] and prospective studies [96] have demonstrated an increased risk of fundic gland polyps associated with long-term PPI use, with similar findings reported for VPZ [97]. PPI/VPZ-associated gastric neuroendocrine tumors appear rare, with evidence limited mainly to case reports. Regarding gastric cancer, observational studies report inconsistent results [98, 99]; confounding factors and reverse causality remain major concerns. Thus, causality has not been established. Nevertheless, long-term PPI/VPZ therapy should be prescribed cautiously, and periodic endoscopy should be considered during prolonged therapy.

### Non-*H. pylori*, non-NSAIDs ulcer

#### CQ-22

How should initial treatment for non-*H. pylori*, non-NSAID peptic ulcers be provided?

- We suggest VPZ or a PPI for the initial treatment of non-*H. pylori*, non-NSAID peptic ulcers.

Recommendation: weak (58.3% agreed for strong, 41.7% agreed for weak recommendation), evidence level: C.

*Comments:* Non-*H. pylori*, non-NSAID peptic ulcers comprise a heterogeneous group of conditions, including Crohn’s disease, gastrin-producing tumors, specific viral or bacterial infections, eosinophilic gastroenteritis, severe physical or psychological stress, and cancer immunotherapy-associated gastritis. Most cases are idiopathic and have a multifactorial pathogenesis in which gastric acid delays mucosal healing [100]. Consequently, potent acid suppression with either VPZ or a PPI is suggested as first-line therapy, although direct head-to-head RCTs are currently lacking. VPZ and PPIs differ in their mechanisms of acid suppression [101]. PPIs require acid-dependent activation and irreversibly inhibit activated H+/K+-ATPase, whereas VPZ is a potassium-competitive acid blocker that rapidly inhibits H+/K+-ATPase without requiring acid activation [101, 102]. VPZ provides more rapid, potent, and sustained acid suppression and is less affected by CYP2C19 polymorphisms than conventional PPIs [12]. However, whether these pharmacological advantages translate into superior healing or recurrence prevention in non-*H. pylori*, non-NSAID peptic ulcers remains unproven. Observational studies have reported healing rates of approximately 70–90% [103–105], with variability likely related to dose and treatment duration [104]. Idiopathic ulcers have lower healing rates than *H. pylori*-associated ulcers, indicating their refractory nature [103, 105].

#### CQ-23

How should the prevention of recurrence be managed after healing of non-*H. pylori*, non-NSAID peptic ulcers?

- We suggest a PPI or an H_2_RA for the prevention of recurrence after the healing of non-*H. pylori*, non-NSAID peptic ulcers.

Recommendation: weak (8.3% agreed for strong, 91.7% agreed for weak recommendation), evidence level: C.

*Comments:* Longitudinal data indicate that untreated non-*H. pylori*, non-NSAID peptic ulcers carry a remarkably high risk of relapse compared with *H. pylori* ulcers [106–108]. Specifically, cumulative recurrence rates without maintenance therapy reach 13.4% at 12 months [106], 13.9% at 18 months in Japan [103], and 16.0–42.3% over long-term follow-up of 5–7 years [107, 108]. Furthermore, non-*H. pylori*, non-NSAID peptic ulcers show significantly higher recurrence rates than with *H. pylori* ulcers after successful eradication [103, 107]. A single double-blind RCT demonstrated that a PPI and a H_2_RA effectively reduced recurrent hemorrhage over 24 months, with no statistically significant difference in efficacy [109]. Moreover, clinical data on potassium-competitive acid blockers, including VPZ, are currently unavailable.

#### CQ-24

How should the treatment of post-bulbar or descending duodenal ulcers be managed?

- As conservative therapy, we suggest VPZ, a PPI, or misoprostol. In refractory cases, we also suggest strategies to reduce mucosal exposure to bile or pancreatic secretions.

Recommendation: weak (91.7% agreed for weak recommendation), evidence level: D.

*Comments:* Post-bulbar duodenal ulcers are rare lesions for which underlying ischemia or malperfusion has been suggested as a major pathological factor [110, 111]. Retrospective cohort studies demonstrated that patients with post-bulbar ulcers have significantly higher rates of severe comorbidities, including advanced age and maintenance dialysis, compared with those with bulbar ulcers [112, 113]. Conservative management generally consists of acid suppression or misoprostol for mucosal protection [113]. In refractory cases, endoscopic biliary or pancreatic duct drainage has been reported to facilitate healing [114]. Because this condition may be associated with a poor prognosis, underlying vascular disorders, such as thrombosis or severe arterial stenosis, should be thoroughly investigated. Interventional radiology or surgery should be considered if clinical exacerbation occurs despite conservative therapy.

#### FRQ-4

What is the frequency of peptic ulcers associated with non-*H. pylori Helicobacter* infection (NHPH)?

- NHPH infection has been detected in some patients with *H. pylori*-negative peptic ulcers, but its frequency remains uncertain.

*Comments:* Studies from Japan, Belgium, and Iran have reported NHPH infection in a subset of patients with peptic ulcer [115–117]. In a Japanese study of *H. pylori*-negative gastroduodenal diseases, NHPH infection was identified in 20.8% of patients, including several with peptic ulcers [115]. However, the true frequency remains unclear because general-population prevalence and optimal diagnostic methods remain uncertain [118]. A multicenter prospective study in Japan reported an NHPH prevalence of 3.0% among health checkup participants [119]. Further development of reliable diagnostic methods [120] is needed to better define the frequency of NHPH-associated peptic ulcers.

### Remnant gastric ulcers

#### CQ-25

How should remnant gastric ulcers be treated?

- PPIs are recommended for the treatment of remnant gastric ulcers.

Recommendation: strong (75.0% agreed for strong recommendation), evidence level: C.

*Comments:* Medical therapy is the first-line treatment for remnant gastric ulcers. In an open-label comparative study, omeprazole showed superior early healing compared with cimetidine, sucralfate, colloidal bismuth, and misoprostol, although differences were no longer significant by 6 weeks. No evidence has shown that VPZ is superior to PPIs; thus, PPIs are recommended, and VPZ may also be considered because of its potent acid-suppressive effect [121].

No RCTs have demonstrated the efficacy of *H. pylori* eradication therapy for remnant gastric ulcers, and observational studies have shown no clear correlation [122–125].

### Therapeutic algorithm

**Fig. 7:**
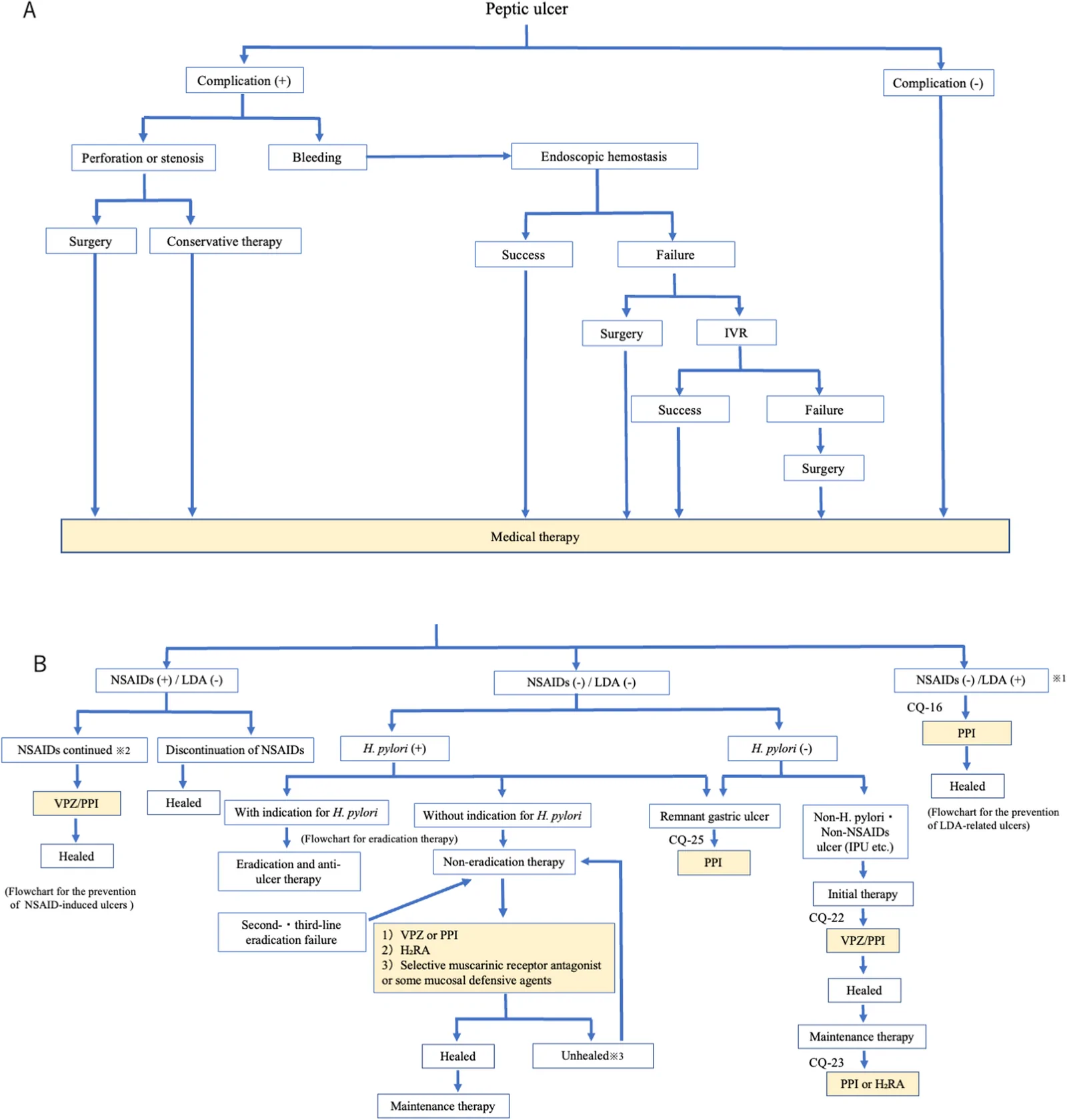
Algorithm for the treatment of peptic ulcer disease. Complicated ulcers are managed first: perforation or stenosis by surgery or conservative therapy, and hemorrhagic ulcers by endoscopic hemostasis, followed by surgery or IVR if hemostasis fails. Uncomplicated ulcers receive immediate medical therapy (**A**). NSAIDs are discontinued, and anti-ulcer therapy is provided for the treatment of NSAID-induced ulcers. If NSAIDs cannot be discontinued, the ulcer should be treated with VPZ or a PPI. LDA-related ulcers should be treated with a PPI without discontinuing LDA whenever possible. Patients with peptic ulcers who do not use NSAIDs or LDA should be tested for *H. pylori*. Eradication therapy is recommended for patients who are *H. pylori* positive. A remnant gastric ulcer is treated with a PPI, and an idiopathic ulcer is treated with a VPZ or a PPI followed by maintenance therapy with a PPI or an H_2_RA (**B**). 1 Discontinuation of LDA should be avoided as much as possible. 2 Contraindications. Administration should be considered only when discontinuation is not possible. 3 Consider the possibility of an idiopathic ulcer. *CQ* clinical question, *H*_*2*_*RA* histamine H2-receptor antagonist, *H. pylori* *Helicobacter pylori*, *IPU* idiopathic peptic ulcer, *IVR* interventional radiology, *LDA* low-dose aspirin, *NSAID* nonsteroidal anti-inflammatory drug, *PPI* proton pump inhibitor, *VPZ* vonoprazan

There are no major changes for therapeutic algorithms in the treatment of peptic ulcers compared with the previous version (Fig. 7A). If complications (e.g., perforation, stenosis, and hemorrhage) are present, they are addressed prior to treatment of the ulcer by surgical and endoscopic treatments and interventional radiology (IVR). When no complications are present, medical therapy is provided immediately.

NSAIDs are discontinued, and a PPI or VPZ is provided for NSAID-induced ulcers. If NSAIDs cannot be discontinued, the ulcer should be treated with a PPI or VPZ. Eradication therapy is recommended for patients with *H. pylori* infection. For patients without an indication for eradication, non-eradication therapy is provided followed by maintenance therapy to prevent ulcer recurrence. A remnant ulcer is treated with a PPI, and an idiopathic ulcer is treated with a PPI or VPZ (Fig. 7B).

**Fig. 8:**
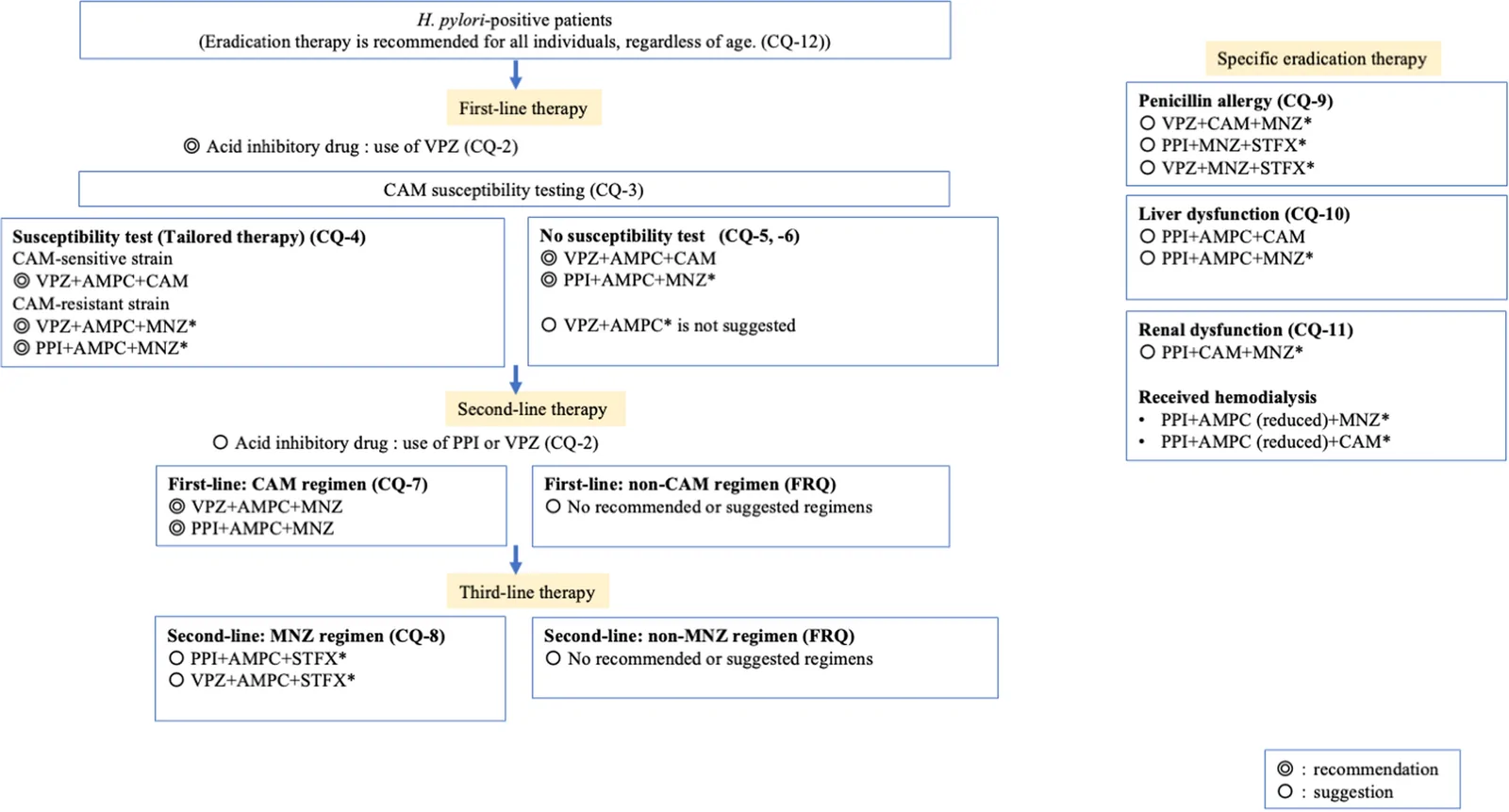
Algorithm for eradication therapy for *H. pylori* infection. Eradication therapy is recommended for all *H. pylori*-positive individuals, regardless of age. If possible, CAM susceptibility testing should be performed before first-line therapy. If first-line therapy fails, second-line therapy is indicated. Specialist consultation is essential for third-line therapy and special clinical situations. MNZ-based first-line regimens are not covered by Japanese health insurance and should be used cautiously because of resistance and safety concerns. Regimens are not covered by the Japanese health insurance system. *AMPC* amoxicillin, *CAM* clarithromycin, *CQ* clinical question, *FRQ* future research question, *H. pylori* *Helicobacter pylori*, *MNZ* metronidazole, *PPI* proton pump inhibitor, *STFX* sitafloxacin, *VPZ* vonoprazan

Regardless of age, individuals with *H. pylori* infection are candidates for eradication therapy, and it is recommended that susceptibility testing for antimicrobial agents be performed prior to treatment (Fig. 8). Regardless of Japanese insurance coverage, Fig. 8 outlines the recommended regimens based on evidence-based medicine (EBM) for various clinical settings, such as first-line, second-line, and third-line therapies, and specific situations (penicillin allergy, and liver and renal dysfunctions).

**Fig. 9:**
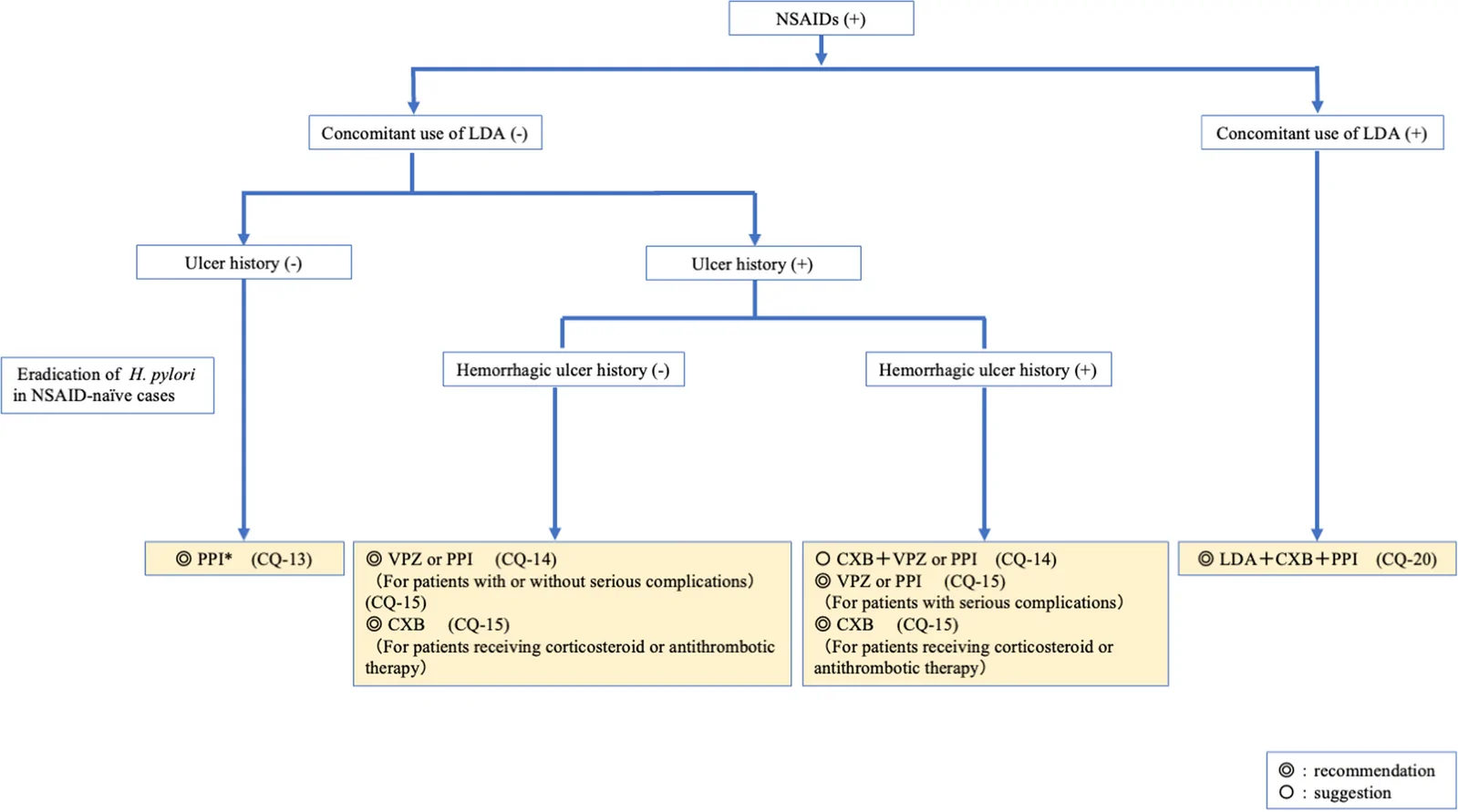
Algorithm for the prevention of NSAID-induced ulcers. Prevention depends on concomitant LDA use, ulcer history, and bleeding history. PPIs are recommended for primary prevention in patients without ulcer history, eradication is recommended for NSAID-naive *H. pylori*-positive patients, and VPZ or a PPI is recommended for recurrence prevention. CXB is recommended in selected high-risk patients and with a PPI when NSAIDs and LDA are combined. Regimens are not covered by Japanese health insurance. *CQ* clinical question, *CXB* celecoxib, *H. pylori* *Helicobacter pylori*, *LDA* low-dose aspirin, *NSAID* nonsteroidal anti-inflammatory drug, *PPI* proton pump inhibitor, *VPZ* vonoprazan

In the algorithm for preventing NSAID-induced ulcers, prevention varies depending on whether LDA is being used concomitantly, whether there is a history of ulcers, and whether there is a history of bleeding ulcers (Fig. 9). For ulcer prevention, a PPI or VPZ should be selected appropriately, and NSAIDs should be switched to celecoxib when indicated. In this section, there are some parts where evidence-based data for VPZ are lacking.

**Fig. 10:**
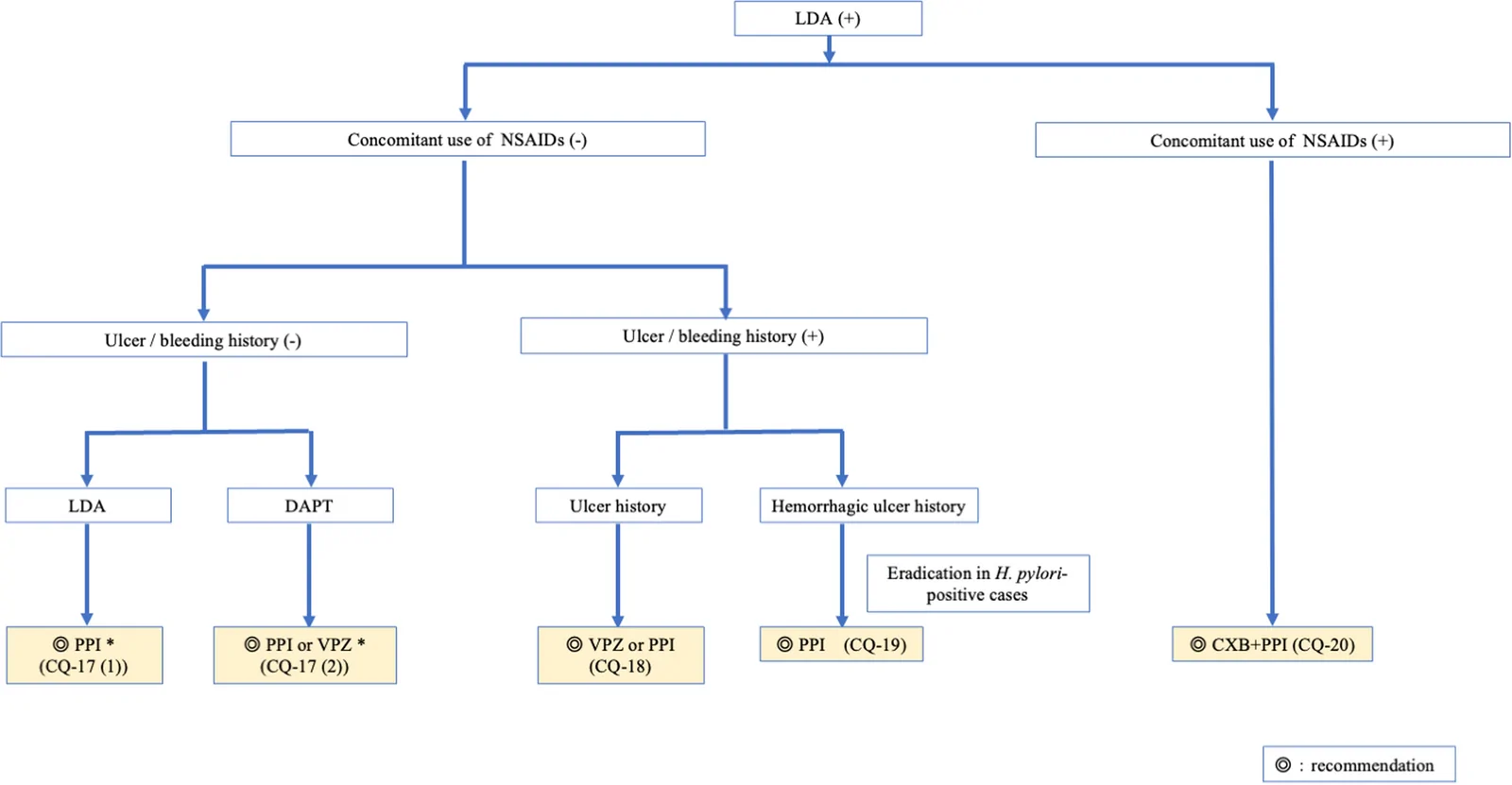
Algorithm for the prevention of LDA-related ulcers. Prevention depends on ulcer/bleeding history and concomitant antiplatelet or NSAID therapy. A PPI is recommended for LDA users without ulcer history, a PPI or VPZ for DAPT users or LDA users with ulcer history, and a PPI for those with hemorrhagic-ulcer history. *H. pylori*-positive patients should receive eradication therapy, and CXB with a PPI is recommended when LDA and NSAIDs are combined. Not covered by Japanese health insurance. *CQ* clinical question, *CXB* celecoxib, *DAPT* dual-antiplatelet therapy, *H. pylori* *Helicobacter pylori*, *LDA* low-dose aspirin, *NSAID* nonsteroidal anti-inflammatory drug, *PPI* proton pump inhibitor, *VPZ* vonoprazan

In the algorithm for preventing LDA-induced ulcers, prevention varies depending on whether NSAIDs are being used concomitantly, whether P2Y12 receptor antagonists are being used concomitantly, and whether there is a history of ulcers or hemorrhage (Fig. 10). In addition to administering eradication therapy to *H. pylori*-positive patients, a PPI or VPZ should be selected appropriately for the prevention of LDA-induced ulcers, and NSAIDs should be switched to celecoxib when they are used concomitantly.

## Appendix

Members of the Guidelines Committee who created and evaluated the JSGE ‘‘Evidence-based clinical practice guidelines for peptic ulcer disease 2026″ are listed below.

## Creation committee

Chair: Tomoari Kamada (Department of Health Care Medicine, Kawasaki Medical School). Vice-Chair: Mitsushige Sugimoto (Division of Genome-Wide Infectious Microbiology, Research Center for GLOBAL and LOCAL Infectious Disease, Oita University). Members: Masanori Ito (Department of General Internal Medicine, Hiroshima University Hospital), Junichi Iwamoto (Department of Gastroenterology, Tokyo Medical University Ibaraki Medical Center), Tadayoshi Okimoto (Department of Gastroenterology, Oita Prefectural Hospital), Shoko Ono (Division of Endoscopy, Hokkaido University Hospital), Takeshi Kanno (Division of Gastroenterology, Tohoku University Graduate School of Medicine/R&D Division of Career Education for Medical Professionals, Medical Education Center, Jichi Medical University), Toshimi Chiba (Division of Internal Medicine, Department of Oral Medicine, Iwate Medical University), Kengo Tokunaga (Department of Preventive Medicine, Kyorin University School of Medicine), Sachiyo Nomura (Department of Gastrointestinal Surgery, Graduate School of Medicine, The University of Tokyo), Takahisa Murao (Department of Health Care Medicine, Kawasaki Medical School), and Toshio Watanabe (Department of Premier Preventive Medicine, Osaka Metropolitan University Graduate School of Medicine).

## SR collaborators

Daisuke Asaoka (Department of Gastroenterology, Juntendo Tokyo Koto Geriatric Medical Center), Eri Iwata (Center for Health Surveillance & Preventive Medicine, Tokyo Medical University Hospital), Ting Wang (Division of Internal Medicine, Department of Oral Medicine, Iwate Medical University), Hideki Ono (Department of Gastroenterology, Oita Prefectural Hospital), Ryo Katsumata (Department of Health Care Medicine, Kawasaki Medical School), Satoshi Kikuchi (Department of Gastroenterology, Sanai Hospital), Naoki Konishi (Department of Gastroenterology, Tokyo Medical University Ibaraki Medical Center), Satoshi Sato (Department of Medicine, Division of Gastroenterology, Jichi Medical University), Aya Sunago (Department of Health Care Medicine, Kawasaki Medical School), Hiromi Sekiguchi (Department of Medicine, Division of Gastroenterology, Jichi Medical University), Hourin Cho (Center for Health Surveillance & Preventive Medicine, Tokyo Medical University Hospital), Daisuke Chinda (Division of Endoscopy, Hirosaki University Hospital), Osamu Dohi (Department of Gastroenterology and Hepatology, Kyoto Prefectural University of Medicine), Akinobu Nakata (Department of Gastroenterology, Osaka Metropolitan University Hospital), Akira Higashimori (Department of Gastroenterology, Naniwaikuno Hospital), Yuka Hirashita (Department of Gastroenterology, Faculty of Medicine, Oita University), Keigo Misawa (Department of Medicine, Division of Gastroenterology, Jichi Medical University), Kazuhiro Mizukami (Department of Gastroenterology, Faculty of Medicine, Oita University), Daisuke Miyamori (Department of General Internal Medicine, Hiroshima University Hospital), Junko Mukohyama (Department of Surgery, The Institute of Medical Science, The University of Tokyo), Hideki Mori (Division of Gastroenterology and Hepatology, Department of Internal Medicine, Keio University School of Medicine), Shuhei Yoshida (Department of General Internal Medicine, Hiroshima University Hospital), and Jun Watanabe (Department of Surgery, Division of Gastroenterological, General and Transplant Surgery, Jichi Medical University).

## Evaluation committee

Chair: Kiichi Sato (Tochigi Medical Examination Plaza). Vice-Chair: Yuji Mizokami (Department of Gastroenterology, New Tokyo Hospital). Member: Atsushi Takagi (Department of Internal Medicine, Kameda Morinosato Hospital).

## The Japanese Society of Gastroenterology

Director General: Satoshi Mochida (Saitama Medical University Hospital); Vice Director General: Susumu Eguchi (Nagasaki University Hospital); Past Vice Director General: Yoshifumi Takeyama (Osaka Gyoumeikan Hospital); Director of the Guidelines Editorial Committee: Takao Itoi (Tokyo Medical University Hospital); Vice Director of the Guidelines Editorial Committee: Hajime Isomoto (Tottori University Hospital); Chairperson of the Guidelines Editorial Committee: Sumio Watanabe (Juntendo University Hospital).

## Cooperative societies

The Japanese Gastroenterological Association, the Japan Gastroenterological Endoscopy Society, and the Japanese Society for Helicobacter Research.

## Abbreviations

AMPC: Amoxicillin
BQs: Background questions
CAM: Clarithromycin
CI: Confidence interval
CQs: Clinical questions
COX: Cyclooxygenase
DAPT: Dual-antiplatelet therapy
FRQs: Future research questions
H_2_RAs: Histamine H2-receptor antagonists
*H. pylori*: *Helicobacter pylori*
ITT: Intention-to-treat
JSGE: Japanese Society of Gastroenterology
LDA: Low-dose aspirin
LVFX: Levofloxacin
MNZ: Metronidazole
NHPH: Non-*H. pylori Helicobacter*
NSAIDs: Nonsteroidal anti-inflammatory drugs
OR: Odds ratio
PP: Per-protocol analysis
PPI: Proton pump inhibitor
RCTs: Randomized controlled trials
SR: Systematic review
STFX: Sitafloxacin
UBT: Urea breath test
UGIB: Upper gastrointestinal bleeding
VPZ: Vonoprazan

## References

[1] Tsuda M, Asaka M, Kato M, Matsushima R, Fujimori K, Akino K, et al. Effect on *Helicobacter pylori* eradication therapy against gastric cancer in Japan. Helicobacter. 2017. 10.1111/hel.12415. PMC5655764 28771894

[2] Kamada T, Satoh K, Itoh T, Ito M, Iwamoto J, Okimoto T, et al. Evidence-based clinical practice guidelines for peptic ulcer disease 2020. J Gastroenterol. 2020;56(4):303–22. 10.1007/s00535-021-01769-0 PMC8005399 33620586

[3] Yoshida M, Kinoshita Y, Watanabe M, Sugano K. JSGE clinical practice guidelines 2014: standards, methods, and process of developing the guidelines. J Gastroenterol. 2014;50(1):4–10. 10.1007/s00535-014-1016-1 25448314

[4] Neumann I, Letelier LM, Rada G, Claro JC, Martin J, Howden CW, et al. Comparison of different regimens of proton pump inhibitors for acute peptic ulcer bleeding. Cochrane Database Syst Rev. 2013;2013(6):CD007999. 10.1002/14651858.CD007999.pub2 PMC10114080 23760821

[5] Jian Z, Li H, Race NS, Ma T, Jin H, Yin Z. Is the era of intravenous proton pump inhibitors coming to an end in patients with bleeding peptic ulcers? Meta-analysis of the published literature. Br J Clin Pharmacol. 2016;82(3):880–9. 10.1111/bcp.12866 PMC5338121 26679691

[6] Sgourakis G, Chatzidakis G, Poulou A, Malliou P, Argyropoulos T, Ravanis G, et al. High-dose vs. low-dose proton pump inhibitors post-endoscopic hemostasis in patients with bleeding peptic ulcer. A meta-analysis and meta-regression analysis. Turk J Gastroenterol. 2018;29(1):22–31. 10.5152/tjg.2018.17143 PMC6322613 29391304

[7] Geeratragool T, Kaosombatwattana U, Boonchote A, Chatthammanat S, Preechakawin N, Srichot J, et al. Comparison of vonoprazan versus intravenous proton pump inhibitor for prevention of high-risk peptic ulcers rebleeding after successful endoscopic hemostasis: a multicenter randomized noninferiority trial. Gastroenterology. 2024;167(4):778-87 e3. 10.1053/j.gastro.2024.03.036 38582271

[8] Pattarapuntakul T, Wong T, Wetwittayakhlang P, Netinatsunton N, Keeratichananont S, Kaewdech A, et al. Efficacy of vonoprazan vs. intravenous proton pump inhibitor in prevention of re-bleeding of high-risk peptic ulcers: a randomized controlled pilot study. J Clin Med. 2024. 10.3390/jcm13123606. PMC11204564 38930134

[9] Sugimoto M, Murata M. Differences and trends in worldwide clinical guidelines for *Helicobacter pylori* infection. Transl Gastroenterol Hepatol. 2026;11:47. 10.21037/tgh-2025-159 PMC13066358 41969561

[10] Ford AC, Delaney BC, Forman D, Moayyedi P. Eradication therapy in *Helicobacter pylori* positive peptic ulcer disease: systematic review and economic analysis. Am J Gastroenterol. 2004;99(9):1833–55. 10.1111/j.1572-0241.2004.40014.x 15330927

[11] Sugimoto M, Furuta T, Shirai N, Kodaira C, Nishino M, Ikuma M, et al. Evidence that the degree and duration of acid suppression are related to *Helicobacter pylori* eradication by triple therapy. Helicobacter. 2007;12(4):317–23. 10.1111/j.1523-5378.2007.00508.x 17669104

[12] Kagami T, Sahara S, Ichikawa H, Uotani T, Yamade M, Sugimoto M, et al. Potent acid inhibition by vonoprazan in comparison with esomeprazole, with reference to CYP2C19 genotype. Aliment Pharmacol Ther. 2016;43(10):1048–59. 10.1111/apt.13588 26991399

[13] Murakami K, Sakurai Y, Shiino M, Funao N, Nishimura A, Asaka M. Vonoprazan, a novel potassium-competitive acid blocker, as a component of first-line and second-line triple therapy for *Helicobacter pylori* eradication: a phase III, randomised, double-blind study. Gut. 2016;65(9):1439–46. 10.1136/gutjnl-2015-311304 PMC5036253 26935876

[14] Sue S, Shibata W, Sasaki T, Kaneko H, Irie K, Kondo M et al (2018) Randomized trial of Vonoprazan- Versus PPI-based third-line triple therapy with sitafloxacin for *Helicobacter pylori*. J Gastroenterol Hepatol 10.1111/jgh.14456 30151994

[15] Hojo M, Asaoka D, Takeda T, Shimada Y, Matsumoto K, Matsumoto K, et al. Randomized controlled study on the effects of triple therapy including vonoprazan or rabeprazole for the second-line treatment of *Helicobacter pylori* infection. Therap Adv Gastroenterol. 2020;13:1756284820966247. 10.1177/1756284820966247 PMC7675913 33240391

[16] Okimoto T, Ando T, Sasaki M, Ono S, Kobayashi I, Shibayama K, et al. Antimicrobial-resistant *Helicobacter pylori* in Japan: report of nationwide surveillance for 2018–2020. Helicobacter. 2024;29(1):e13028. 10.1111/hel.13028 37823466

[17] Ma Q, Li H, Liao J, Cai Z, Zhang B. Tailored therapy for *Helicobacter pylori* eradication: a systematic review and meta-analysis. Front Pharmacol. 2022;13:908202. 10.3389/fphar.2022.908202 PMC9495299 36160444

[18] Tsuda M, Watanabe Y, Oikawa R, Watanabe R, Higashino M, Kubo K, et al. Clinical evaluation of a novel molecular diagnosis kit for detecting *Helicobacter pylori* and clarithromycin-resistant using intragastric fluid. Helicobacter. 2022;27(6):e12933. 10.1111/hel.12933 PMC9788249 36263754

[19] Isomoto H, Shimoyama T, Ito M, Osaki T, Toyoshima O, Matsuzaki J, et al. Guidelines for the management of *Helicobacter pylori* infection in Japan: 2024 revised edition. J Gastroenterol. 2024. 10.1007/s00535-026-02379-4. PMC13283202 41902905

[20] Kawai T, Yamagishi T, Yagi K, Kataoka M, Kawakami K, Sofuni A, et al. Tailored eradication therapy based on fecal *Helicobacter pylori* clarithromycin sensitivities. J Gastroenterol Hepatol. 2008;23(Suppl 2):S171–4. 10.1111/j.1440-1746.2008.05408.x 19120893

[21] Lee HJ, Kim JI, Cheung DY, Kim TH, Jun EJ, Oh JH, et al. Eradication of *Helicobacter pylori* according to 23S ribosomal RNA point mutations associated with clarithromycin resistance. J Infect Dis. 2013;208(7):1123–30. 10.1093/infdis/jit287 23801607

[22] Park CS, Lee SM, Park CH, Koh HR, Jun CH, Park SY, et al. Pretreatment antimicrobial susceptibility-guided vs. clarithromycin-based triple therapy for *Helicobacter pylori* eradication in a region with high rates of multiple drug resistance. Am J Gastroenterol. 2014;109(10):1595–602. 10.1038/ajg.2014.222 25091062

[23] Hsieh MS, Kuo FC, Wu MC, Wang JW, Liu CJ, Chu NS, et al. Tailored susceptibility-guided therapy via gastric juice PCR for the first-line *H. pylori* eradication, a randomized controlled trial. J Formos Med Assoc. 2022;121(8):1450–7. 10.1016/j.jfma.2021.10.011 34836662

[24] Liu L, Shi H, Shi Y, Wang A, Guo N, Li F, et al. Vonoprazan-based therapies versus PPI-based therapies in patients with H. pylori infection: systematic review and meta-analyses of randomized controlled trials. Helicobacter. 2024;29(3):e13094. 10.1111/hel.13094 38790090

[25] Murata M, Sugimoto M, Mizuno H, Kanno T, Satoh K. Clarithromycin versus metronidazole in first-line *Helicobacter pylori* triple eradication therapy based on resistance to antimicrobial agents: meta-analysis. J Clin Med. 2020. 10.3390/jcm9020543. PMC7073899 32079208

[26] Suzuki S, Gotoda T, Kusano C, Ikehara H, Ichijima R, Ohyauchi M, et al. Seven-day vonoprazan and low-dose amoxicillin dual therapy as first-line *Helicobacter pylori* treatment: a multicentre randomised trial in Japan. Gut. 2020;69(6):1019–26. 10.1136/gutjnl-2019-319954 PMC7282559 31915235

[27] Chey WD, Megraud F, Laine L, Lopez LJ, Hunt BJ, Howden CW. Vonoprazan triple and dual therapy for *Helicobacter pylori* infection in the United States and Europe: randomized clinical trial. Gastroenterology. 2022;163(3):608–19. 10.1053/j.gastro.2022.05.055 35679950

[28] Furuta T, Yamade M, Higuchi T, Takahashi S, Ishida N, Tani S, et al. Expectations for the dual therapy with vonoprazan and amoxicillin for the eradication of *H. pylori*. J Clin Med. 2023. 10.3390/jcm12093110. PMC10179648 37176551

[29] Shinozaki S, Kobayashi Y, Osawa H, Sakamoto H, Hayashi Y, Lefor AK, et al. Effectiveness and safety of vonoprazan versus proton pump inhibitors for second-line *Helicobacter pylori* eradication therapy: systematic review and meta-analysis. Digestion. 2021;102(3):319–25. 10.1159/000504939 31914442

[30] Murakami K, Furuta T, Ando T, Nakajima T, Inui Y, Oshima T, et al. Multi-center randomized controlled study to establish the standard third-line regimen for *Helicobacter pylori* eradication in Japan. J Gastroenterol. 2013;48(10):1128–35. 10.1007/s00535-012-0731-8 23307042

[31] Furuta T, Sugimoto M, Kodaira C, Nishino M, Yamade M, Uotani T, et al. Sitafloxacin-based third-line rescue regimens for *Helicobacter pylori* infection in Japan. J Gastroenterol Hepatol. 2014;29(3):487–93. 10.1111/jgh.12442 24224808

[32] Mori H, Suzuki H, Matsuzaki J, Tsugawa H, Fukuhara S, Miyoshi S, et al. Efficacy of 10-day sitafloxacin-containing third-line rescue therapies for *Helicobacter pylori* strains containing the *gyrA* mutation. Helicobacter. 2016;21(4):286–94. 10.1111/hel.12286 26612407

[33] Mori H, Suzuki H, Matsuzaki J, Tsugawa H, Fukuhara S, Miyoshi S, et al. Rifabutin-based 10-day and 14-day triple therapy as a third-line and fourth-line regimen for *Helicobacter pylori* eradication: a pilot study. United Eur Gastroenterol J. 2016;4(3):380–7. 10.1177/2050640615618043 PMC4924440 27403304

[34] Sue S, Shibata W, Sasaki T, Kaneko H, Irie K, Kondo M, et al. Randomized trial of vonoprazan-based versus proton-pump inhibitor-based third-line triple therapy with sitafloxacin for *Helicobacter pylori*. J Gastroenterol Hepatol. 2019;34(4):686–92. 10.1111/jgh.14456 30151994

[35] Furuta T, Baba S, Yamade M, Uotani T, Kagami T, Suzuki T, et al. High incidence of autoimmune gastritis in patients misdiagnosed with two or more failures of *H. pylori* eradication. Aliment Pharmacol Ther. 2018;48(3):370–7. 10.1111/apt.14849 29920721

[36] Ono S, Kato M, Nakagawa S, Mabe K, Sakamoto N. Vonoprazan improves the efficacy of *Helicobacter pylori* eradication therapy with a regimen consisting of clarithromycin and metronidazole in patients allergic to penicillin. Helicobacter. 2017. 10.1111/hel.12374. 28098408

[37] Sue S, Suzuki N, Shibata W, Sasaki T, Yamada H, Kaneko H, et al. First-line *Helicobacter pylori* eradication with vonoprazan, clarithromycin, and metronidazole in patients allergic to penicillin. Gastroenterol Res Pract. 2017. 10.1155/2017/2019802. PMC5664290 29181022

[38] Sue S, Sasaki T, Kaneko H, Irie K, Kondo M, Maeda S. *Helicobacter pylori* rescue treatment with vonoprazan, metronidazole, and sitafloxacin in the presence of penicillin allergy. JGH Open. 2021;5(2):307–11. 10.1002/jgh3.12492 PMC7857288 33553672

[39] Furuta T, Sugimoto M, Yamade M, Uotani T, Sahara S, Ichikawa H, et al. Eradication of *H. pylori* infection in patients allergic to penicillin using triple therapy with a PPI, metronidazole and sitafloxacin. Intern Med. 2014;53(6):571–5. 10.2169/internalmedicine.53.1677 24633026

[40] Mori H, Suzuki H, Matsuzaki J, Masaoka T, Kanai T. Antibiotic resistance and *gyrA* mutation affect the efficacy of 10-day sitafloxacin-metronidazole-esomeprazole therapy for *Helicobacter pylori* in penicillin allergic patients. United Eur Gastroenterol J. 2017;5(6):796–804. 10.1177/2050640616688995 PMC5625875 29026593

[41] Azuma T, Ito S, Suto H, Ito Y, Miyaji H, Yamazaki Y, et al. Pharmacokinetics of clarithromycin in *Helicobacter pylori* eradication therapy in patients with liver cirrhosis. Aliment Pharmacol Ther. 2000;14(Suppl 1):216–22. 10.1046/j.1365-2036.2000.014s1216.x 10807427

[42] Furusyo N, Walaa AH, Eiraku K, Toyoda K, Ogawa E, Ikezaki H, et al. Treatment for eradication of *Helicobacter pylori* infection among chronic hepatitis C patients. Gut Liver. 2011;5(4):447–53. 10.5009/gnl.2011.5.4.447 PMC3240787 22195242

[43] Tsai CE, Liang CM, Lee CH, Kuo YH, Wu KL, Chiu YC, et al. First-line *Helicobacter pylori* eradication among patients with chronic liver diseases in Taiwan. Kaohsiung J Med Sci. 2016;32(8):397–402. 10.1016/j.kjms.2016.05.012 PMC11915914 27523452

[44] Sheu BS, Huang JJ, Yang HB, Huang AH, Wu JJ. The selection of triple therapy for *Helicobacter pylori* eradication in chronic renal insufficiency. Aliment Pharmacol Ther. 2003;17(10):1283–90. 10.1046/j.1365-2036.2003.01527.x 12755841

[45] Chang WC, Jo YI, Park HS, Jegal J, Park JH, Lee JH, et al. *Helicobacter pylori* eradication with a 7-day low-dose triple therapy in hemodialysis patients. Clin Exp Nephrol. 2010;14(5):469–73. 10.1007/s10157-010-0319-7 20632062

[46] Seyyedmajidi M, Falaknazi K, Mirsattari D, Zojaji H, Roshani M, Lahmi F, et al. Correlation between creatinine clearance and *Helicobacter pylori* infection eradication with sequential and triple therapeutic regimens: a randomised clinical trial. Arab J Gastroenterol. 2011;12(3):150–3. 10.1016/j.ajg.2011.07.004 22055594

[47] Liang CM, Chiu CH, Wang HM, Tai WC, Yao CC, Tsai CE, et al. First-line *Helicobacter pylori* eradication in patients with chronic kidney diseases in Taiwan. Biomed Res Int. 2017;2017:3762194. 10.1155/2017/3762194 PMC5742431 29376072

[48] Sahara S, Sugimoto M, Ichikawa H, Kagami T, Sakao Y, Ohashi N, et al. Efficacy of reduced dosage of amoxicillin in an eradication therapy for *Helicobacter pylori* infection in patients on hemodialysis: a randomized controlled trial. Digestion. 2018;97(2):163–9. 10.1159/000484981 29310119

[49] Pilotto A, Franceschi M, Di Mario F, Leandro G, Bozzola L, Valerio G. The long-term clinical outcome of elderly patients with *Helicobacter pylori*-associated peptic ulcer disease. Gerontology. 1998;44(3):153–8. 10.1159/000022000 9592687

[50] Gong H, Xu HM, Zhang DK. Focusing on *Helicobacter pylori* infection in the elderly. Front Cell Infect Microbiol. 2023;13:1121947. 10.3389/fcimb.2023.1121947 PMC10036784 36968116

[51] Iwata E, Sugimoto M, Asaoka D, Hojo M, Ito M, Kitazawa N, et al. Characteristics of *Helicobacter pylori* eradication therapy in patients 80 years or Older living in a metropolitan area: a multicenter retrospective study. Helicobacter. 2024;29(4):13125. 10.1111/hel.13125 39152662

[52] Kobayashi S, Joshita S, Yamamoto C, Yanagisawa T, Miyazawa T, Miyazawa M, et al. Efficacy and safety of eradication therapy for elderly patients with *Helicobacter pylori* infection. Medicine (Baltimore). 2019;98(30):e16619. 10.1097/MD.0000000000016619 PMC6709141 31348311

[53] Ye Q, Shao X, Shen R, Chen D, Shen J. Changes in the human gut microbiota composition caused by *Helicobacter pylori* eradication therapy: a systematic review and meta-analysis. Helicobacter. 2020;25(4):e12713. 10.1111/hel.12713 32515529

[54] Horii T, Suzuki S, Takano C, Shibuya H, Ichijima R, Kusano C, et al. Lower impact of vonoprazan-amoxicillin dual therapy on gut microbiota for *Helicobacter pylori* eradication. J Gastroenterol Hepatol. 2021. 10.1111/jgh.15572. 34107551

[55] Chen J, Zhang Y, Min H, Zhi J, Ma S, Dong H, et al. Dynamic changes in the gut microbiota after bismuth quadruple therapy and high-dose dual therapy for *Helicobacter pylori* eradication. Helicobacter. 2024;29(2):e13077. 10.1111/hel.13077 38682268

[56] Guan JL, Xu TT, Lin Y, Mo YS, He BY, Han YY, et al. High-dose dual therapy for *Helicobacter pylori* eradication inducing less impact on the gut microbiota. Gut Pathog. 2025;17(1):7. 10.1186/s13099-025-00682-8 PMC11783801 39885529

[57] Bai XF, Tian D, Wang TY, Shu JC, He YJ, Zhu MJ. The impact of probiotics on gut microbiota in the eradication of *Helicobacter pylori* infection: a systematic review. Eur Rev Med Pharmacol Sci. 2023;27(14):6736–43. 10.26355/eurrev_202307_33144 37522685

[58] Koch M. Non-steroidal anti-inflammatory drug gastropathy: clinical results with misoprostol. Ital J Gastroenterol Hepatol. 1999;31(Suppl 1):S54-62. 10379471

[59] Bianchi Porro G, Lazzaroni M, Petrillo M, Manzionna G, Montrone F, Caruso I. Prevention of gastroduodenal damage with omeprazole in patients receiving continuous NSAIDs treatment. A double blind placebo controlled study. Ital J Gastroenterol Hepatol. 1998;30(1):43–7. 9615264

[60] Taha AS, Hudson N, Hawkey CJ, Swannell AJ, Trye PN, Cottrell J, et al. Famotidine for the prevention of gastric and duodenal ulcers caused by nonsteroidal antiinflammatory drugs. N Engl J Med. 1996;334(22):1435–9. 10.1056/NEJM199605303342204 8618582

[61] Scally B, Emberson JR, Spata E, Reith C, Davies K, Halls H, et al. Effects of gastroprotectant drugs for the prevention and treatment of peptic ulcer disease and its complications: a meta-analysis of randomised trials. Lancet Gastroenterol Hepatol. 2018;3(4):231–41. 10.1016/S2468-1253(18)30037-2 PMC5842491 29475806

[62] Koch M, Dezi A, Tarquini M, Capurso L. Prevention of non-steroidal anti-inflammatory drug-induced gastrointestinal mucosal injury: risk factors for serious complications. Dig Liver Dis. 2000;32(2):138–51. 10.1016/s1590-8658(00)80402-8 10975790

[63] Sugano K, Kinoshita Y, Miwa H, Takeuchi T, Esomeprazole NPSG. Randomised clinical trial: esomeprazole for the prevention of nonsteroidal anti-inflammatory drug-related peptic ulcers in Japanese patients. Aliment Pharmacol Ther. 2012;36(2):115–25. 10.1111/j.1365-2036.2012.05133.x 22591121

[64] Sugano K, Kontani T, Katsuo S, Takei Y, Sakaki N, Ashida K, et al. Lansoprazole for secondary prevention of gastric or duodenal ulcers associated with long-term non-steroidal anti-inflammatory drug (NSAID) therapy: results of a prospective, multicenter, double-blind, randomized, double-dummy, active-controlled trial. J Gastroenterol. 2012;47(5):540–52. 10.1007/s00535-012-0541-z PMC3360874 22388884

[65] Chan FK, Wong VW, Suen BY, Wu JC, Ching JY, Hung LC, et al. Combination of a cyclo-oxygenase-2 inhibitor and a proton-pump inhibitor for prevention of recurrent ulcer bleeding in patients at very high risk: a double-blind, randomised trial. Lancet. 2007;369(9573):1621–6. 10.1016/S0140-6736(07)60749-1 17499604

[66] Mizokami Y, Oda K, Funao N, Nishimura A, Soen S, Kawai T, et al. Vonoprazan prevents ulcer recurrence during long-term NSAID therapy: randomised, lansoprazole-controlled non-inferiority and single-blind extension study. Gut. 2018;67(6):1042–51. 10.1136/gutjnl-2017-314010 PMC5969369 28988197

[67] Kawai T, Suzuki C, Honda Y, Fernandez JL. Long-term safety and effectiveness of vonoprazan for prevention of gastric and duodenal ulcer recurrence in patients on nonsteroidal anti-inflammatory drugs in Japan: a 12-month post-marketing surveillance study. Expert Opin Drug Saf. 2023;22(5):425–31. 10.1080/14740338.2023.2136163 36264125

[68] Masclee GM, Valkhoff VE, Coloma PM, de Ridder M, Romio S, Schuemie MJ, et al. Risk of upper gastrointestinal bleeding from different drug combinations. Gastroenterology. 2014;147(4):784-92 e9 (**quiz e13–4**). 10.1053/j.gastro.2014.06.007 24937265

[69] Vonkeman HE, Fernandes RW, van der Palen J, van Roon EN, van de Laar MA. Proton-pump inhibitors are associated with a reduced risk for bleeding and perforated gastroduodenal ulcers attributable to non-steroidal anti-inflammatory drugs: a nested case-control study. Arthritis Res Ther. 2007;9(3):R52. 10.1186/ar2207 PMC2206342 17521422

[70] Sung JJ, Lau JY, Ching JY, Wu JC, Lee YT, Chiu PW, et al. Continuation of low-dose aspirin therapy in peptic ulcer bleeding: a randomized trial. Ann Intern Med. 2010;152(1):1–9. 10.7326/0003-4819-152-1-201001050-00179 19949136

[71] Liu CP, Chen WC, Lai KH, Mar GY, Lin SY, Ger LP, et al. Esomeprazole alone compared with esomeprazole plus aspirin for the treatment of aspirin-related peptic ulcers. Am J Gastroenterol. 2012;107(7):1022–9. 10.1038/ajg.2012.87 22508148

[72] Yeomans N, Lanas A, Labenz J, van Zanten SV, van Rensburg C, Racz I, et al. Efficacy of esomeprazole (20 mg once daily) for reducing the risk of gastroduodenal ulcers associated with continuous use of low-dose aspirin. Am J Gastroenterol. 2008;103(10):2465–73. 10.1111/j.1572-0241.2008.01995.x 18637091

[73] Scheiman JM, Devereaux PJ, Herlitz J, Katelaris PH, Lanas A, Veldhuyzen van Zanten S, et al. Prevention of peptic ulcers with esomeprazole in patients at risk of ulcer development treated with low-dose acetylsalicylic acid: a randomised, controlled trial (OBERON). Heart. 2011;97(10):797–802. 10.1136/hrt.2010.217547 PMC3088470 21415072

[74] Moayyedi P, Eikelboom JW, Bosch J, Connolly SJ, Dyal L, Shestakovska O, et al. Pantoprazole to prevent gastroduodenal events in patients receiving rivaroxaban and/or aspirin in a randomized, double-blind placebo-controlled trial. Gastroenterology. 2019;157(2):403–125. 10.1053/j.gastro.2019.04.041 31054846

[75] Tunggal P, Ng FH, Lam KF, Chan FK, Lau YK. Effect of esomeprazole versus famotidine on platelet inhibition by clopidogrel: a double-blind, randomized trial. Am Heart J. 2011;162(5):870–4. 10.1016/j.ahj.2011.08.007 22093203

[76] Ng FH, Tunggal P, Chu WM, Lam KF, Li A, Chan K, et al. Esomeprazole compared with famotidine in the prevention of upper gastrointestinal bleeding in patients with acute coronary syndrome or myocardial infarction. Am J Gastroenterol. 2012;107(3):389–96. 10.1038/ajg.2011.385 22108447

[77] Yano H, Tsukahara K, Morita S, Endo T, Sugano T, Hibi K, et al. Influence of omeprazole and famotidine on the antiplatelet effects of clopidogrel in addition to aspirin in patients with acute coronary syndromes: a prospective, randomized, multicenter study. Circ J. 2012;76(11):2673–80. 10.1253/circj.cj-12-0511 22864179

[78] Tsujita K, Deguchi H, Uda A, Sugano K. Upper gastrointestinal bleeding in Japanese patients with ischemic heart disease receiving vonoprazan or a proton pump inhibitor with multiple antithrombotic agents: a nationwide database study. J Cardiol. 2020;76(1):51–7. 10.1016/j.jjcc.2020.02.012 32184027

[79] Sugano K, Matsumoto Y, Itabashi T, Abe S, Sakaki N, Ashida K, et al. Lansoprazole for secondary prevention of gastric or duodenal ulcers associated with long-term low-dose aspirin therapy: results of a prospective, multicenter, double-blind, randomized, double-dummy, active-controlled trial. J Gastroenterol. 2011;46(6):724–35. 10.1007/s00535-011-0397-7 PMC3117278 21499703

[80] Sanuki T, Fujita T, Kutsumi H, Hayakumo T, Yoshida S, Inokuchi H, et al. Rabeprazole reduces the recurrence risk of peptic ulcers associated with low-dose aspirin in patients with cardiovascular or cerebrovascular disease: a prospective randomized active-controlled trial. J Gastroenterol. 2012;47(11):1186–97. 10.1007/s00535-012-0588-x 22526273

[81] Iwakiri R, Higuchi K, Kato M, Fujishiro M, Kinoshita Y, Watanabe T, et al. Randomised clinical trial: prevention of recurrence of peptic ulcers by rabeprazole in patients taking low-dose aspirin. Aliment Pharmacol Ther. 2014;40(7):780–95. 10.1111/apt.12907 25100080

[82] Sugano K, Choi MG, Lin JT, Goto S, Okada Y, Kinoshita Y, et al. Multinational, double-blind, randomised, placebo-controlled, prospective study of esomeprazole in the prevention of recurrent peptic ulcer in low-dose acetylsalicylic acid users: the LAVENDER study. Gut. 2014;63(7):1061–8. 10.1136/gutjnl-2013-304722 24326741

[83] Ng FH, Wong SY, Lam KF, Chu WM, Chan P, Ling YH, et al. Famotidine is inferior to pantoprazole in preventing recurrence of aspirin-related peptic ulcers or erosions. Gastroenterology. 2010;138(1):82–8. 10.1053/j.gastro.2009.09.063 19837071

[84] Chan FK, Kyaw M, Tanigawa T, Higuchi K, Fujimoto K, Cheong PK, et al. Similar efficacy of proton-pump inhibitors vs H2-Receptor antagonists in reducing risk of upper gastrointestinal bleeding or ulcers in high-risk users of low-dose aspirin. Gastroenterology. 2017;152(1):105-10 e1. 10.1053/j.gastro.2016.09.006 27641510

[85] Tseng ZF, Hsu PI, Peng NJ, Kao SS, Tsay FW, Cheng JS, et al. Omeprazole vs famotidine for the prevention of gastroduodenal injury in high-risk users of low-dose aspirin: a randomized controlled trial. J Chin Med Assoc. 2021;84(1):19–24. 10.1097/JCMA.0000000000000465 PMC12965974 33230059

[86] Kawai T, Oda K, Funao N, Nishimura A, Matsumoto Y, Mizokami Y, et al. Vonoprazan prevents low-dose aspirin-associated ulcer recurrence: randomised phase 3 study. Gut. 2018;67(6):1033–41. 10.1136/gutjnl-2017-314852 PMC5969345 29196436

[87] Lai KC, Lam SK, Chu KM, Wong BC, Hui WM, Hu WH, et al. Lansoprazole for the prevention of recurrences of ulcer complications from long-term low-dose aspirin use. N Engl J Med. 2002;346(26):2033–8. 10.1056/NEJMoa012877 12087138

[88] Goldstein JL, Huang B, Amer F, Christopoulos NG. Ulcer recurrence in high-risk patients receiving nonsteroidalanti-inflammatory drugs plus low-dose aspirin: results of a post HOC subanalysis. Clin Ther. 2004;26(10):1637–43. 10.1016/j.clinthera.2004.10.002 15598480

[89] Goldstein JL, Hochberg MC, Fort JG, Zhang Y, Hwang C, Sostek M. Clinical trial: the incidence of NSAID-associated endoscopic gastric ulcers in patients treated with PN 400 (naproxen plus esomeprazole magnesium) vs. enteric-coated naproxen alone. Aliment Pharmacol Ther. 2010;32(3):401–13. 10.1111/j.1365-2036.2010.04378.x 20497139

[90] Goldstein JL, Lowry SC, Lanza FL, Schwartz HI, Dodge WE. The impact of low-dose aspirin on endoscopic gastric and duodenal ulcer rates in users of a non-selective non-steroidal anti-inflammatory drug or a cyclo-oxygenase-2-selective inhibitor. Aliment Pharmacol Ther. 2006;23(10):1489–98. 10.1111/j.1365-2036.2006.02912.x 16669964

[91] Strand V. Are COX-2 inhibitors preferable to non-selective non-steroidal anti-inflammatory drugs in patients with risk of cardiovascular events taking low-dose aspirin? Lancet. 2007;370(9605):2138–51. 10.1016/S0140-6736(07)61909-6 18156036

[92] Lee SR, Kwon S, Choi EK, Jung JH, Han KD, Oh S, et al. Proton pump inhibitor co-therapy in patients with atrial fibrillation treated with oral anticoagulants and a prior history of upper gastrointestinal tract bleeding. Cardiovasc Drugs Ther. 2022;36(4):679–89. 10.1007/s10557-021-07170-6 33730289

[93] Ray WA, Chung CP, Murray KT, Smalley WE, Daugherty JR, Dupont WD, et al. Association of oral anticoagulants and proton pump inhibitor cotherapy with hospitalization for upper gastrointestinal tract bleeding. JAMA. 2018;320(21):2221–30. 10.1001/jama.2018.17242 PMC6404233 30512099

[94] Gralnek IM, Stanley AJ, Morris AJ, Camus M, Lau J, Lanas A, et al. Endoscopic diagnosis and management of nonvariceal upper gastrointestinal hemorrhage (NVUGIH): European Society of Gastrointestinal Endoscopy (ESGE) guideline - update 2021. Endoscopy. 2021;53(3):300–32. 10.1055/a-1369-5274 33567467

[95] Martin FC, Chenevix-Trench G, Yeomans ND. Systematic review with meta-analysis: fundic gland polyps and proton pump inhibitors. Aliment Pharmacol Ther. 2016;44(9):915–25. 10.1111/apt.13800 27634363

[96] Hongo M, Fujimoto K, Gastric Polyps Study G. Incidence and risk factor of fundic gland polyp and hyperplastic polyp in long-term proton pump inhibitor therapy: a prospective study in Japan. J Gastroenterol. 2010;45(6):618–24. 10.1007/s00535-010-0207-7 20177714

[97] Uemura N, Kinoshita Y, Haruma K, Kushima R, Yao T, Akiyama J, et al. Vonoprazan as a long-term maintenance treatment for erosive esophagitis: VISION, a 5-year, randomized, open-label study. Clin Gastroenterol Hepatol. 2025;23(5):748-57 e5. 10.1016/j.cgh.2024.08.004 39209187

[98] Peng TR, Wu TW, Li CH. Association between proton-pump inhibitors and the risk of gastric cancer: a systematic review and meta-analysis. Int J Clin Oncol. 2023;28(1):99–109. 10.1007/s10147-022-02253-2 36224477

[99] Piovani D, Tsantes AG, Schunemann HJ, Bonovas S. Meta-analysis: use of proton pump inhibitors and risk of gastric cancer in patients requiring gastric acid suppression. Aliment Pharmacol Ther. 2023;57(6):653–65. 10.1111/apt.17360 36585832

[100] McColl KE, El-Nujumi AM, Chittajallu RS, Dahill SW, Dorrian CA, El-Omar E, et al. A study of the pathogenesis of *Helicobacter pylori* negative chronic duodenal ulceration. Gut. 1993;34(6):762–8. 10.1136/gut.34.6.762 PMC1374258 8314508

[101] Echizen H. The first-in-class potassium-competitive acid blocker, vonoprazan fumarate: pharmacokinetic and pharmacodynamic considerations. Clin Pharmacokinet. 2016;55(4):409–18. 10.1007/s40262-015-0326-7 26369775

[102] Thuy HM, Sugimoto M, Quang PMN, Yamaoka Y. CYP2C19 genotype-guided dosing of proton pump inhibitors: progress in clinical trials and real-world use. Expert Rev Clin Pharmacol. 2026;19(2):133–50. 10.1080/17512433.2026.2626454 41618926

[103] Kanno T, Iijima K, Abe Y, Yagi M, Asonuma S, Ohyauchi M, et al. *Helicobacter pylori*-negative and non-steroidal anti-inflammatory drugs-negative idiopathic peptic ulcers show refractoriness and high recurrence incidence: multicenter follow-up study of peptic ulcers in Japan. Dig Endosc. 2016;28(5):556–63. 10.1111/den.12635 26866510

[104] Dore MP, Soro S, Niolu C, Longo NP, Bibbo S, Manca A, et al. Clinical features and natural history of idiopathic peptic ulcers: a retrospective case-control study. Scand J Gastroenterol. 2019;54(11):1315–21. 10.1080/00365521.2019.1679247 31630582

[105] Sugawara K, Koizumi S, Horikawa Y, Mimori N, Tsuji T, Ishii H, et al. Is the new potent acid-inhibitory drug vonoprazan effective for healing idiopathic peptic ulcers? A multicenter observational study in Akita Prefecture, Japan. J Gastroenterol. 2019;54(11):963–71. 10.1007/s00535-019-01587-5 31037448

[106] Hung LC, Ching JY, Sung JJ, To KF, Hui AJ, Wong VW, et al. Long-term outcome of *Helicobacter pylori*-negative idiopathic bleeding ulcers: a prospective cohort study. Gastroenterology. 2005;128(7):1845–50. 10.1053/j.gastro.2005.03.026 15940620

[107] Wong GL, Wong VW, Chan Y, Ching JY, Au K, Hui AJ, et al. High incidence of mortality and recurrent bleeding in patients with *Helicobacter pylori*-negative idiopathic bleeding ulcers. Gastroenterology. 2009;137(2):525–31. 10.1053/j.gastro.2009.05.006 19445937

[108] Yoon H, Kim SG, Jung HC, Song IS. High recurrence rate of idiopathic peptic ulcers in long-term follow-up. Gut Liver. 2013;7(2):175–81. 10.5009/gnl.2013.7.2.175 PMC3607771 23560153

[109] Wong GLH, Lau LHS, Ching JYL, Tse YK, Ling RHY, Wong VWS, et al. Prevention of recurrent idiopathic gastroduodenal ulcer bleeding: a double-blind, randomised trial. Gut. 2020;69(4):652–7. 10.1136/gutjnl-2019-318715 31229990

[110] Jang ES, Jeong SH, Kim JW, Lee SH, Yoon CJ, Kang SG. A case of acute ischemic duodenal ulcer associated with superior mesenteric artery dissection after transarterial chemoembolization for hepatocellular carcinoma. Cardiovasc Intervent Radiol. 2009;32(2):367–70. 10.1007/s00270-008-9442-1 18931873

[111] Peixoto A, Goncalves R, Silva M, Paiva D, Lopes J, Macedo G. Extensive ulcerative duodenitis caused by ischemia. Clin Res Hepatol Gastroenterol. 2017;41(2):119–20. 10.1016/j.clinre.2016.06.004 27459878

[112] Matsuhashi T, Fukuda S, Abe Y, Mikami T, Tatsuta T, Hikichi T, et al. Nature and treatment outcomes of bleeding post-bulbar duodenal ulcers. Dig Endosc. 2022;34(5):984–93. 10.1111/den.14160 34609030

[113] Sekiguchi H, Shinozaki S, Takezawa T, Osawa H, Miura Y, Lefor AK, et al. Long-term outcomes in patients with post-bulbar ulcer bleeding compared to bulbar ulcer bleeding in the duodenum. Digestion. 2022;103(2):126–32. 10.1159/000519293 34551417

[114] Shoji A, Shichijo S, Fukutake N. Endoscopic naso-biliary and -pancreatic duct drainage for refractory post-bulbar duodenal ulcer bleeding. Dig Endosc. 2020;32(6):999. 10.1111/den.13781 32578242

[115] Nakamura M, Overby A, Michimae H, Matsui H, Takahashi S, Mabe K, et al. PCR analysis and specific immunohistochemistry revealing a high prevalence of non-Helicobacter pylori Helicobacters in Helicobacter pylori-negative gastric disease patients in Japan: high susceptibility to an Hp eradication regimen. Helicobacter. 2020;25(5):e12700. 10.1111/hel.12700 32790220

[116] Shafaie S, Kaboosi H, Peyravii Ghadikolaii F. Prevalence of non *Helicobacter pylori* gastric *Helicobacters* in Iranian dyspeptic patients. BMC Gastroenterol. 2020;20(1):190. 10.1186/s12876-020-01331-x PMC7298804 32546214

[117] Taillieu E, De Witte C, De Schepper H, Van Moerkercke W, Rutten S, Michiels S, et al. Clinical significance and impact of gastric non-*Helicobacter pyloriHelicobacter* species in gastric disease. Aliment Pharmacol Ther. 2023;57(12):1432–44. 10.1111/apt.17488 36975151

[118] Sugimoto M, Rimbara E, Murata M, Yamaoka Y. Roles of gastric non-*Helicobacter pyloriHelicobacter* species in gastric disease development: a review. Helicobacter. 2025;30(3):e70051. 10.1111/hel.70051 40462295

[119] Tokunaga K, Rimbara E, Tsukadaira T, Mabe K, Yahara K, Suzuki H, et al. Prospective multicenter surveillance of non-*H. pylori* Helicobacter infections during medical checkups, Japan. Emerg Infect Dis. 2025;31(6):1121–30. 10.3201/eid3106.241315 PMC12123907 40439414

[120] Matsui H, Rimbara E, Suzuki M, Tokunaga K, Suzuki H, Sano M, et al. Development of serological assays to identify *Helicobacter suis* and *H. pylori* infections. iScience. 2023;26(4):106522. 10.1016/j.isci.2023.106522 PMC10139984 37123222

[121] Janke A, Stasiewicz J, Namiot Z, Szalaj W. Treatment of the gastric stump ulcer: an open study with five drugs. Hepatogastroenterology. 2000;47(34):1195–8. 11020913

[122] Leivonen MK, Haglund CH, Nordling SF. *Helicobacter pylori* infection after partial gastrectomy for peptic ulcer and its role in relapsing disease. Eur J Gastroenterol Hepatol. 1997;9(4):371–4. 9160200

[123] Lee YT, Sung JJ, Choi CL, Chan FK, Ng EK, Ching JY, et al. Ulcer recurrence after gastric surgery: is *Helicobacter pylori* the culprit? Am J Gastroenterol. 1998;93(6):928–31. 10.1111/j.1572-0241.1998.00279.x 9647021

[124] Schilling D, Adamek HE, Wilke J, Schauwecker P, Martin WR, Arnold JC, et al. Prevalence and clinical importance of Helicobacter pylori infection in patients after partial gastric resection for peptic ulcer disease. A prospective evaluation of Helicobacter pylori infection on 50 resected patients compared with matched nonresected controls. Z Gastroenterol. 1999;37(2):127–32. 10190245

[125] Huang WH, Wang HH, Wu WW, Lai HC, Hsu CH, Cheng KS. Helicobacter pylori infection in patients with ulcer recurrence after partial gastrectomy. Hepatogastroenterology. 2004;51(59):1551–3. 15362799

